# Structural basis of liver de-targeting and neuronal tropism of CNS-targeted AAV capsids

**DOI:** 10.1016/j.ymthe.2026.03.030

**Published:** 2026-03-28

**Authors:** Tyler J Brittain, Seongmin Jang, Gerard M Coughlin, Jonathan D Hoang, Bre’Anna H Barcelona, Izabela Giriat, Fiona Ristic, Nathan Appling, Camille PMA Chossis, Timothy F Shay, Viviana Gradinaru

**Affiliations:** 1 Division of Biology & Biological Engineering, California Institute of Technology, Pasadena CA 91125, USA; 2 Howard Hughes Medical Institute, Caltech, Pasadena, CA 91125, USA

**Keywords:** AAV, AAVR, PKD2, blood-brain barrier, directed evolution, brain, liver

## Abstract

Developing effective vectors for gene therapy requires accurate on-target coverage while minimizing off-target transduction that can lead to adverse events. In mice, the engineered capsid PHP.eB shows enhanced brain transduction, while the further engineered CAP-B10 is also de-targeted from astrocytes and liver. Here, we solved cryo-EM structures of CAP-B10 and its complex with AAV receptor (AAVR) domain PKD2, at 2.22 and 2.20 Å resolutions, respectively. These structures reveal a motif that hinders AAVR binding, which we confirmed by measuring affinities. We showed that this motif is transferable to other capsids by solving cryo-EM structures of AAV9-X1, at 3.09 Å, and AAV9-X1.1 without and with PKD2, at 2.51 and 2.18 Å, respectively. Using this structural information, we designed and validated novel AAV variants with reduced liver and altered brain cell tropism *in vivo*. Overall, we provide a framework for using structural information to guide rational engineering of gene delivery vectors to achieve safe and effective delivery.

## Graphical Abstract

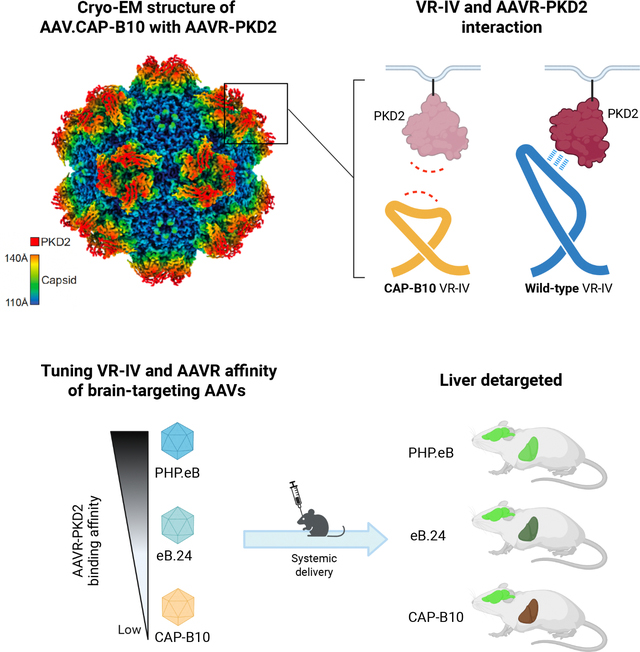

Gradinaru and colleagues explore the structural basis of the tropism of several AAV gene delivery vectors engineered by directed evolution to cross the blood-brain barrier after systemic administration. They identify a critical receptor-binding motif that can guide rational AAV engineering for safer gene therapy for diseases of the brain.

## Introduction

Effective gene therapy for neurological diseases requires gene delivery vectors capable of mediating sufficient transgene expression in the central nervous system (CNS) while avoiding immunogenicity^1–4^. Adeno-associated viruses (AAV) are FDA approved for the treatment of CNS diseases due to their low pathogenicity and stable gene expression across broad cell types including neurons^5–10^. The AAV9 serotype has been of particular interest in engineering due to its natural ability to penetrate the blood-brain barrier (BBB) after systemic (intravenous) delivery^11,12^. However, AAV9 and other naturally-occurring AAVs show poor efficiency in crossing the BBB and transduce many tissues body-wide. Notably, transduction of the liver by AAVs can lead to hepatotoxicity, a highly problematic adverse event^13–17^. This limits the potential application of natural AAV vectors as vehicles for brain gene therapy.

Directed evolution studies have shown that the tropism profiles of AAV capsids are modifiable, suggesting that engineered vectors could bypass the efficiency and safety issues of naturally-evolved AAV vectors^18,19^. Indeed, high-throughput screening strategies have yielded multiple engineered capsids that have been widely adopted in both basic and preclinical research. AAV9-derived capsid variant libraries are commonly made by inserting a random 7-mer sequence between residues 588 and 589 of the surface-exposed variable region (VR)-VIII^20–22^. *In vivo* selections with this library design yielded the brain-enhanced capsid AAV-PHP.B^21^ and subsequent engineering of flanking amino acids yielded the now-widely-used AAV-PHP.eB (PHP.eB)^23^, which is highly enriched in the CNS and moderately de-targeted from other organs in mice after intravenous administration. Additional engineering of PHP.eB at VR-IV by substitution of residues 452–458 with a randomized 7-mer identified a further improved version, AAV.CAP-B10 (CAP-B10)^24^, which more effectively de-targets from the liver in mice. Furthermore, CAP-B10 shows a neuron-specific tropism with less astrocytic transduction in the mouse CNS, suggesting that VR-IV may also influence brain cell type tropism. A separate VR-VIII library selection found the capsid AAV9-X1, which is highly endothelial specific in the rodent brain^25^. By adopting the same VR-IV substitution of CAP-B10, AAV9-X1 was further improved to AAV9-X1.1, which exhibits better production yield and stability, while maintaining a similar specificity for brain endothelial cells and a comparable level of liver transduction. However, it remains unclear why the VR-IV substitution of CAP-B10 within AAV9-X1.1 does not confer liver de-targeting on AAV9-X1.1.

These variants were optimized in mice; the design of similarly enhanced vectors for clinical application is hindered by our limited understanding of AAV infection mechanisms across organs and species, with only a few naturally-evolved receptor-AAV interactions identified to date. For example, terminal N-linked galactose on the cell surface is well known as a primary attachment factor for AAV9 and its derivatives ^26–28^. Another crucial factor is the AAV receptor (AAVR)^29–31^, AAVR is expressed on the cell surface across various organs and tissues^32–36^. The plasma membrane population is small and rapidly recycled, with most AAVR found in the trans-Golgi network^29^. Recent work suggests that its role also extend beyond AAV internalization^30^. AAVR comprises five repeated immunoglobulin (Ig)-like polycystic kidney disease domains (PKD1–5), all sharing a conserved β-barrel structure. Each PKD domain presents distinct residues on its surface, facilitating unique interactions. For instance, among PKD1–5, PKD2 exclusively interacts with AAV9^22^, via a binding interface spanning many capsid regions.

Current engineering efforts aim to guide AAVs away from the liver, primarily focusing on ablating capsid glycan binding, which decreases basal AAV infectivity across organs^27,37,38^. However, some of these modifications also make AAV transduction-deficient, stressing the importance of a trade-off between off-target reduction and on-target efficiency. Recent structural studies from our group and others showed that the moderately liver de-targeted PHP.eB has reduced affinity for PKD2 compared to AAV9^22,39^. Cryo-electron microscopy (cryo-EM) structures of PHP.eB alone^22^ and in complex with PKD2^39^ revealed that PHP.eB gained a new hydrophilic interaction between residues in VR-VIII, which we termed the “lysine lever,” that rigidly orients the loop; we suggest that this causes a steric clash with AAVR-PKD2^22^. This observation suggests that liver transduction may be governed by AAVR-PKD2 affinity.

In this study, we trace the directed-evolution path of AAVs from AAV9 to PHP.eB to CAP-B10, the most liver de-targeted variant in its lineage to date, using structural and biophysical methodologies. We present seven cryo-EM structures of CNS-targeting and/or liver de-targeted AAVs, along with their AAVR binding affinities. Based on the resulting structural insights, we rationally designed novel AAVs with limited liver transduction. Together, our results reveal the mechanism by which directed evolution has changed receptor affinity and transduction selectivity, which should aid in the rational design of engineered AAVs with enhanced liver de-targeting.

## Results

### The VR-IV modification of CAP-B10 disrupts interaction with AAVR-PKD2

**Figure 1:**
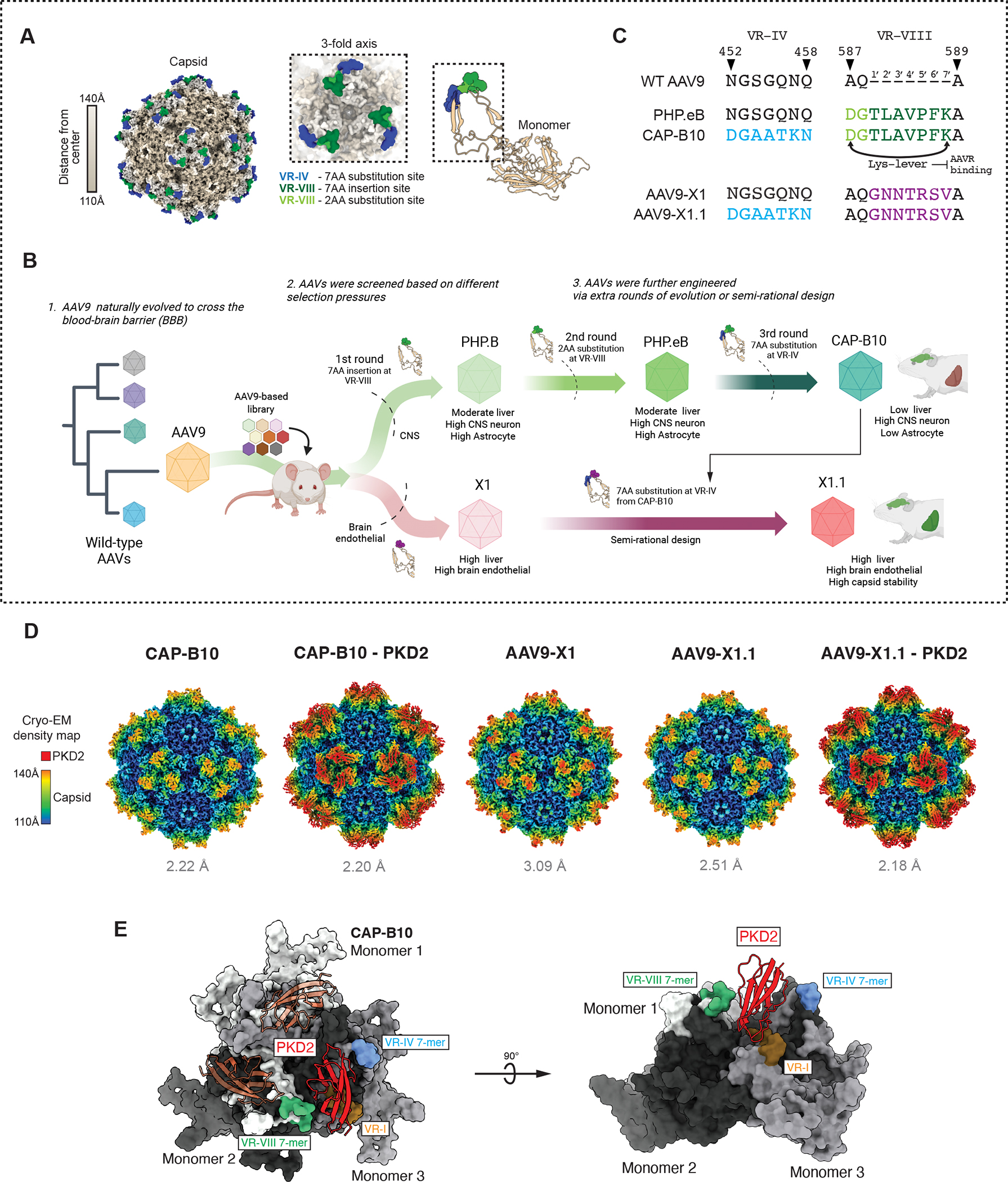
Structure of engineered CNS-enhanced AAVs. **(A)** Location of engineered sites on the AAV capsid shown on structure of PHP.eB (PDB: 7UD4^21^). **(B)** Schematic of directed evolution efforts yielding capsids with high transduction of rodent CNS and low transduction of liver (PHP.B, PHP.eB, and CAP-B10; green arrows), and high transduction of brain endothelial cells and liver (AAV9-X1 and AAV9-X1.1; red arrows). **(C)** Sequence alignment of VR-IV and VR-VIII regions in engineered capsids. The CAP-B10 7-mer substitution is shown in blue, the PHP.eB 7-mer insertion in dark green, two point mutations in light green, and the AAV9-X1 7-mer insertion in purple. D587 and K7’ in PHP.eB and CAP-B10 form the “lysine lever” which diminishes AAVR-PKD2 binding. **(D)** Single-particle cryo-EM maps of engineered capsids with and without PKD2, the AAV9-binding domain of AAVR. In the maps, color indicates distance from the center of the capsid. **(E)** Binding interface between PKD2 and 3-fold face of AAV. AAVR-PKD2 is shown in red, VR-I in orange, 7-mer substitution in VR-IV in blue, and 7-mer insertion in VR-VIII in green. The individual monomers of the AAV 3-fold face are colored white, light gray, and dark gray.

The brain-enhanced capsids PHP.eB and CAP-B10 were sequentially derived from AAV9 along a directed evolution path (Figures 1A–1C, Table 1). This generated CAP-B10 from PHP.eB, which introduced neuronal cellular specificity and enhanced liver de-targeting. The underlying mechanism is unclear, but a clue may be inferred from the structure of PHP.eB in complex with its receptor^39^, showing how a “lysine lever” interaction in the VR-VIII 7-mer introduces a steric clash that partially destabilizes the capsid’s interaction with AAVR-PKD2 (Figures 1C and S1A)^22^. Extending this observation may help explain CAP-B10’s significantly lower liver infectivity. We hypothesized that the VR-IV 7-mer mutation in CAP-B10 (^452^NGSGQNQ^458^ to ^452^DGAATKN^458^) may further disrupt AAVR-PKD2 binding, and thereby further reduce off-target, especially liver, transduction. To investigate whether the VR-IV mutation in CAP-B10 impacts AAVR affinity, we determined high-resolution cryo-EM structures of three capsids: CAP-B10 (2.22 Å resolution), AAV9-X1 (3.09 Å) and AAV9-X1.1 (2.51 Å). We also solved the structures of CAP-B10 and AAV9-X1.1, which share the same VR-IV sequence motif, in complex with AAVR-PKD2 (2.20 and 2.18 Å, respectively) (Figures 1D, S2, S3, Table S1). In both structures of receptor-capsid complexes, PKD2 domains are associated with the capsid, binding the plateaus around the 3-fold axis, as seen previously in PKD2-complexed structures of AAV9 and PHP.eB^20,22,39^. PKD2 appears to engage the VR-VIII, VR-IV, and VR-I loops of the capsid as a binding platform (Figures 1E and S1B). We did not observe a difference in PKD2 position between AAV9-X1.1 and CAP-B10, indicating that the 7-residue difference in VR-VIII does not significantly alter the general binding interface.

**Figure 2:**
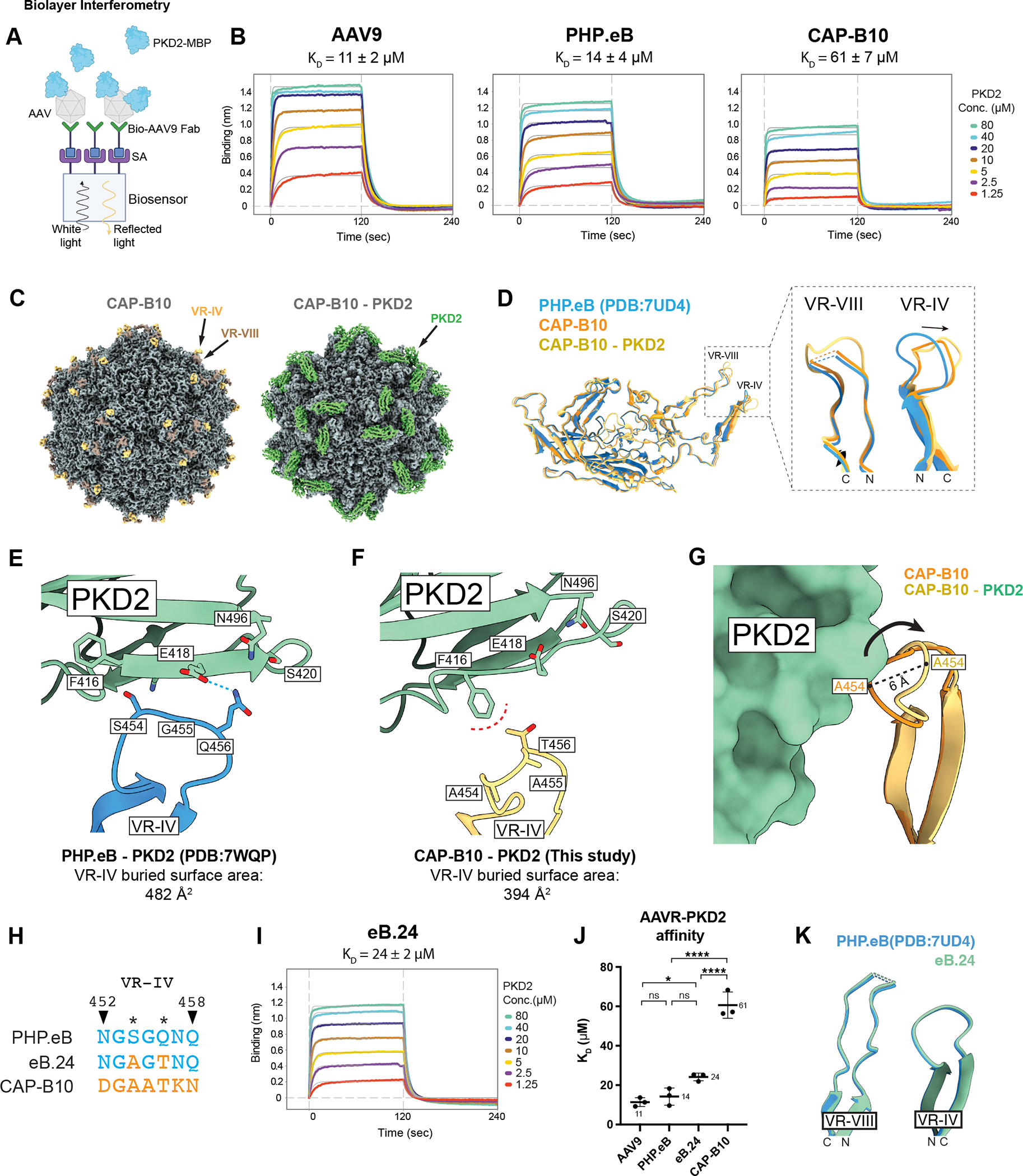
VR-IV modification in CAP-B10 reduces AAVR-PKD2 affinity. **(A)** Schematic of biolayer interferometry (BLI) to measure the binding affinity between AAV capsids and AAVR-PKD2 domain, tagged with high molecular-weight maltose binding protein (MBP) to maximize signal relative to noise. Biotinylated AAV9 affinity ligand (Bio-AAV9 Fab) was immobilized on a streptavidin (SA)-coated biosensor, AAVs loaded as the analyte, and binding quantified by sensorgrams. **(B)** Representative sensorgrams of interactions between AAV9, PHP.eB, and CAP-B10 with AAVR-PKD2. Assays were performed three times, with one representative dataset shown. The averaged equilibrium dissociation constant (Kd) is presented for each capsid. **(C)** Cryo-EM maps of CAP-B10 without and with PKD2. In the uncomplexed map, VR-IV density is shown in orange and VR-VIII density in brown. In the complex map, PKD2 density is shown in green. **(D)** Atomic models of the VP3 subunit of PHP.eB (PDB: 7UD4), CAP-B10 (this study), and CAP-B10 complexed with PKD2 (this study), overlaid to show structural differences and similarities. Enlargement shows (left) that the structure of VR-VIII in PHP.eB and CAP-B10 is similar, and in the presence of PKD2 additional residues become resolvable, and (right) VR-IV loop structural differences between CAP-B10 (orange) and PHP.eB (blue). Arrow indicates the lower position of VR-IV in CAP-B10 relative to PHP.eB, and the additional conformational change when CAP-B10 is bound to PKD2 (yellow). **(E)** PHP.eB has favorable amino acids at positions 456 and 454 to interact with PKD2. Q456 on PHP.eB can form a hydrogen bond to PKD2 E418 and N496, with PKD2 S420 and PHP.eB S454 aiding in creating a hydrophilic environment. Bulky F416 on PKD2 is positioned away from these hydrophilic residues thereby preventing steric clashes. Dashed sky blue line indicates potential hydrogen bonding. VR-IV (residues 450–460) buried surface area was calculated to be 482 Å^2^ at the PKD2 VR-IV interface. **(F)** Our CAP-B10 PKD2 complex structure reveals that two mutations, S454A and Q456T, play a key role in disrupting the PKD2 interaction. Short T456 prevents hydrophilic interaction with E418, N496, and S420 on PKD2. A454 sterically clashes with F416 on PKD2, causing the VR-IV loop to bend away from PKD2. In addition to these two residues, the generally downwardly bent structure of VR-IV further distances T456 from hydrophilic PKD2 residues. VR-IV(residues 450–460) buried surface area was calculated to be 394 Å^2^ at the PKD2 VR-IV interface. **(G)** Comparison of structures of CAP-B10 (orange) and CAP-B10 complexed with PKD2 (yellow) shows that PKD2 induces a conformational change in the VR-IV of CAP-B10. The greatest shift occurs at A454, which is shifted 6 Å farther away from PKD2. **(H)** VR-IV sequences of PHP.eB, eB.24, and CAP-B10. The two point mutations in eB.24, S454A and Q456T, are highlighted in orange. **(I)** Representative BLI sensorgram of eB.24 binding of AAVR-PKD2. **(J)** Affinity of engineered capsids for AAVR-PKD2. Kd values are an average of 3 replicates. **(K)** Cryo-EM-based atomic model of eB.24 overlaid with the structure of PHP.eB (PDB: 7UD4). The two point mutations (S454A, Q456T) in eB.24 do not modify the VR-IV backbone structure of PHP.eB. Statistical significance was determined using ANOVA. *p < 0.05, **p < 0.01, ***p < 0.001, and ****p < 0.0001, ns not significant.

Since our structures confirmed that the VR-IV loop, mutated in CAP-B10 and AAV9-X1.1, is closely apposed to AAVR-PKD2, we next compared the binding affinity of CAP-B10 for AAVR-PKD2 with that of its ancestral capsids, AAV9 and PHP.eB, measuring equilibrium dissociation constants (K_d_) by biolayer interferometry (BLI) (Figure 2A, Table S2). AAV9 and derivatives were loaded onto the BLI biosensor and PKD2, serving as the binding analyte, was introduced to the sensor surface at varying concentrations. CAP-B10 interacted with AAVR-PKD2 with a K_d_ of 61 μM, indicating a weaker binding affinity than PHP.eB (K_d_: 14 μM) (Figure 2B). The wild-type AAV9 exhibited a robust binding affinity (K_d_: 11 μM), consistent with previously reported findings^22^. These results confirm that the 7-residue substitution in the VR-IV region of CAP-B10 disrupts its interaction with AAVR-PKD2.

### Cryo-EM structure of CAP-B10 complexed with AAVR-PKD2 reveals VR-IV interaction with PKD2

To gain detailed structural insight into the mechanism responsible for the reduced CAP-B10 interaction with PKD2, we delved into our cryo-EM structures of CAP-B10 (Figures 2C-G). Electron density was sufficiently resolved to allow us to fit a model for the protein backbone and all but the most solvent-exposed sidechains, for which lack of density is most likely due to flexibility in sidechain positions^40,41^ (Figures S2B, S2C). Although PKD2 density was more disordered and had lower resolution than the overall complex, we were able to fit the PKD2 backbone and all sidechains except for the presumably flexible residues E418, S420, L451, E453, I456, and S457 (Figures S2A, S2D). We first analyzed structural differences in the VR-IV loop of CAP-B10 (^452^DGAATKN^458^) compared to AAV9 and PHP.eB (^452^NGSGQNQ^458^). Compared to PHP.eB, the VR-IV loop of CAP-B10 is angled away from the capsid surface (Figure 2D). A previously-solved structure of the PHP.eB–PKD2 complex showed that the VR-IV loop contributes to the interaction interface (Figure 2E). The PKD2 interaction surface is polar and acidic, and the wild-type VR-IV in PHP.eB accesses this region through polar residues. Electron density reveals that PKD2 residues E418, S420 and N496 participate in an extended polar interaction network (Figure S4A, **right**) and an electrostatic potential map of the interaction site reveals complementary charge surfaces across the interface between PKD2 and PHP.eB VR-IV (Figure S4B). Additionally, the bulky F416 on PKD2 is angled away from PHP.eB S454 in the structure, avoiding any steric hindrance and helping the VR-IV loop of PHP.eB form a stable binding interface with PKD2. Q456 of PHP.eB is positioned such that it may form a hydrogen bond with E418 on PKD2, a hypothesis supported by the electron density of these residues when viewed at a lower threshold and the postulated bond length being within the accepted range for hydrogen bonds (Figure S4A). This polar interface is disrupted in CAP-B10 (discussed below), which has weaker PKD2 binding affinity, leading us to believe that this interface may contribute to the stability of the PHP.eB–PKD2 interaction (Figures 2E and S4A, B).

Our CAP-B10–PKD2 complex structure shows that the polar interface with wild-type VR-IV in PHP.eB is severely disturbed (Figures 2D, 2F, and S4C). The angled-away backbone of the CAP-B10 VR-IV loop reduces the buried surface area in the AAV–AAVR interface from 482 Å^2^ (PHP.eB) to 394 Å^2^ (CAP-B10) (Figures 2E, 2F, S4A-C). Based on the structures, we hypothesize that two residues in the CAP-B10 VR-IV substitution may be instrumental in this reduction of the binding interface. CAP-B10’s Q456T mutation precludes the putative hydrogen bonding interaction with PKD2, and S454A replaces a polar serine with a hydrophobic alanine, allowing F416 on PKD2 to rotate into the polar interface and further disrupt the polar interaction network (Figures 2D, 2F, and S4C). These changes necessitate a large ~6 Å conformational shift of the VR-IV loop backbone when transitioning to the PKD2-complexed form. (Figure 2G).

To test the hypothesized impact of the S454A and Q456T mutations on PKD2 binding, we created a variant capsid (named eB.24) with only these two residues mutated from the PHP.eB sequence (^452^NG**A**G**T**NQ^458^, bold residues indicate mutations). By BLI, eB.24 showed significantly stronger binding affinity for PKD2 (K_d_ 24 μM) compared to CAP-B10, and significantly weaker binding affinity than AAV9. By rank order, the measured affinity of eB.24 falls in between those of PHP.eB and CAP-B10 (Figures 2H–2J). To explain the intermediate level of PKD2 binding in eB.24, we solved a cryo-EM structure of eB.24 at a resolution of 2.05 Å (Figures 2K, S2, S4D-S4F) and compared its structural features with those of PHP.eB and CAP-B10. This comparison revealed that the backbone structures of VR-IV and VR-VIII in eB.24 are nearly identical to those in PHP.eB (Figure 2K), indicating that the two point mutations at residues 454 and 456 are not sufficient to alter the VR-IV backbone conformation and do not affect the structure of VR-VIII. These findings suggest that the difference in PKD2 affinity between PHP.eB and CAP-B10 results from alterations in the overall loop conformation rather than disrupted side chain interactions of amino acids 454 and 456 alone.

We next focused on VR-VIII of CAP-B10, which retains the 7-mer insertion and two point mutations (^587^DGTLAVPFK^7’^) from PHP.eB (we denote the amino acids in the CAP-B10 and PHP.eB 7-mer as T1’, L2’, A3’, V4’, P5’, F6’, and K7’). As expected, the VR-VIII loop of CAP-B10 exhibits a downwardly bent structure similar to that observed in PHP.eB (Figures 2D and S1A). As in PHP.eB, an electrostatic interaction formed by D587 and K7’ induces inward tension that bends the 7-mer downward toward the capsid surface. As discussed above, this causes a steric clash with PKD2, with the hydrophobic patch ^2’^LAV^4’^ oriented toward the hydrophilic surface of PKD2, thereby plausibly reducing AAVR binding. CAP-B10 therefore incorporates two features that together explain its even weaker affinity for AAVR-PKD2: 1) the sterically hindering loop of VR-VIII inherited from PHP.eB and 2) the less hydrophilic loop of VR-IV unique to CAP-B10.

Next, we examined whether the VR-IV modification impacts other previously-identified PHP.eB and AAV9 receptors, such as LY6A and D-galactose. We performed pull-down assays, using agarose beads charged with each respective receptor as bait, and analyzed the amount of AAV captured (Figure S5A). As a control, we first tested the interactions of AAV9, PHP.eB, eB.24, and CAP-B10 with AAVR-PKD2 to validate the consistency of this method with BLI results (Figures S5B and S5C). As expected, AAV9 showed the highest PKD2 capture efficiency, while CAP-B10 captured markedly less PKD2, confirming that the pull-down assay results align with BLI results.

Next, we assessed LY6A and D-galactose binding (Figures S5D and S5E). Unlike AAVR-PKD2, there was no large difference in receptor binding affinity among PHP.eB, eB.24, and CAP-B10. We observed that CAP-B10 was captured by D-galactose slightly less than PHP.eB or eB.24, but this trend was much less pronounced than the decreasing pattern observed in PKD2 binding from PHP.eB to CAP-B10. This suggests that the CAP-B10 structural motif selectively alters PKD2 affinity while not altering other interactions. It follows then that the tropism differences between PHP.eB and CAP-B10 are primarily driven by the difference in PKD2 affinity, since LY6A and D-galactose interactions remain largely unchanged.

### AAV9-X1.1 VR-VIII counteracts diminished AAVR binding mediated by the CAP-B10 VR-IV motif

**Figure 3:**
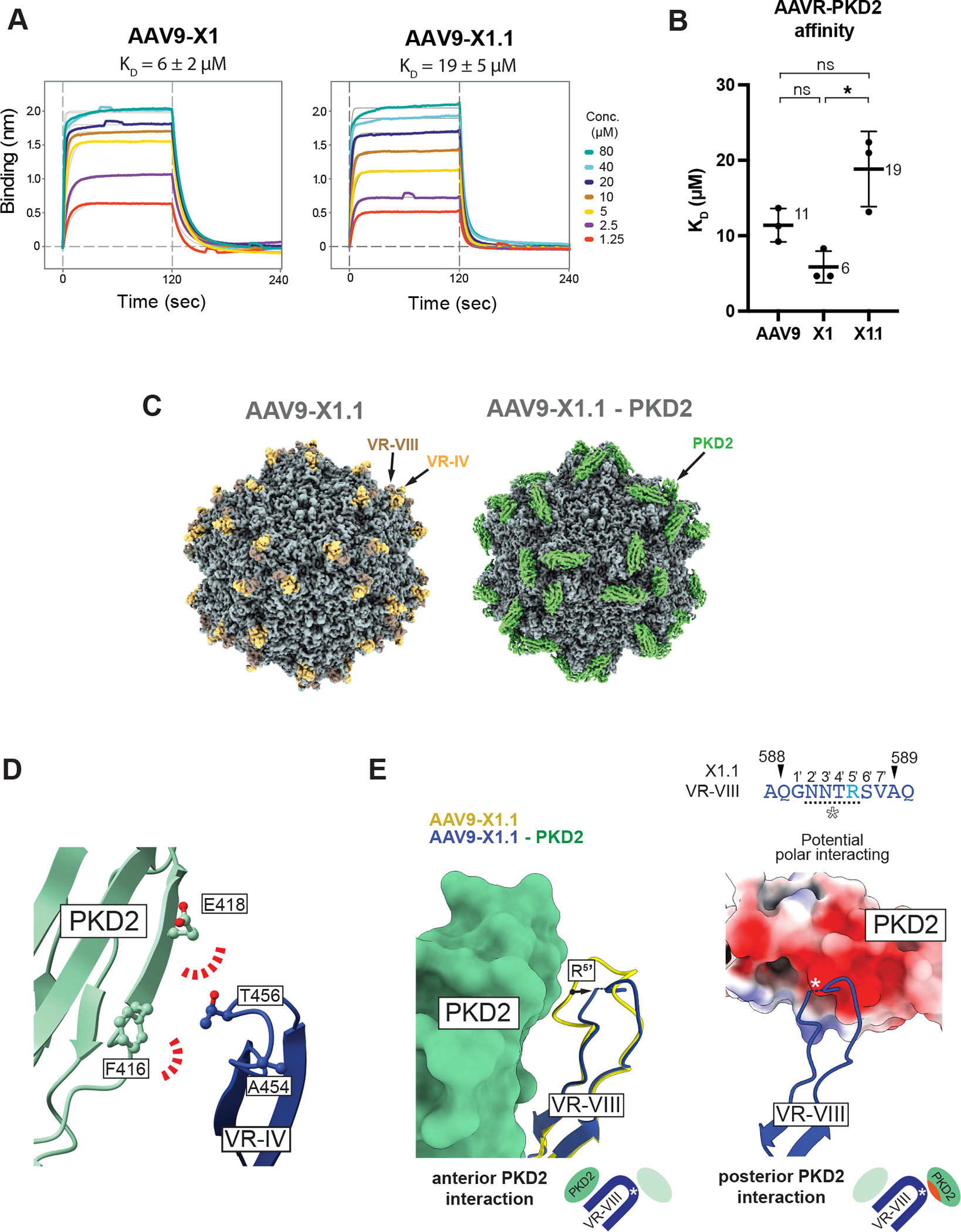
Structural and biophysical characterization of AAV9-X1 and AAV9-X1.1 binding to AAVR-PKD2 **(A)** Representative BLI sensorgrams showing the interaction of AAV9-X1 and AAV9-X1.1 with AAVR-PKD2. Kd values are averages of three replicates. **(B)** Comparison of AAVR-PKD2 binding affinity of AAV9, AAV9-X1, and AAV9-X1.1. AAV9-X1.1 shows weaker AAVR-PKD2 binding affinity compared to AAV9-X1, indicating that the CAP-B10 VR-IV structural motif introduced in AAV9-X1.1 reduces AAVR-PKD2 binding, similarly to CAP-B10. Note that the AAV9 - AAVR-PKD2 affinity shown here is from the same experiment as in Figure 2J. **(C)** Cryo-EM maps of AAV9-X1.1 in its unbound and AAVR-PKD2 bound states. In the uncomplexed structure, the VR-IV is shown in orange and VR-VIII is in brown. In the complex map, PKD2 density is highlighted in green. **(D)** Atomic model of VR-IV in AAV9-X1.1 (blue) complexed with PKD2 (green). As in CAP-B10, T456 loses its putative hydrogen bond to PKD2 E418, while A454 is shifted away from PKD2 and F416 on PKD2 shifts into the PKD2–VR-IV interface. **(E)** Atomic model of VR-VIII in AAV9-X1.1 (blue) complexed with PKD2 (green surface), overlaid with AAV9-X1.1 (yellow). The schematics below illustrate the interactions between PKD2 and VR-VIII of AAV9-X1.1. Left: Interaction of VR-VIII with the anterior PKD2. Right: Interaction of VR-VIII with the posterior PKD2. Asterisks denote the four polar-basic amino acids (^2’^NNTR^5’^) at the protruding end of the X1 7-mer that potentially interact with the polar-acidic surface of PKD2. Statistical significance was determined using ANOVA. *p < 0.05, ns not significant.

After confirming the AAVR hindrance effect of the CAP-B10 7-mer, we investigated whether this property could be transferred to other capsids. To test this, we examined AAV9-X1 and AAV9-X1.1, which selectively target endothelial cells in the rodent brain (Figure 1). Unlike CAP-B10, these vectors target the CNS through the LRP6 receptor which, unlike LY6A, is also expressed in the liver^34,35,42–44^, where it contributes to vector transduction^45^. Since liver de-targeting was not selected for during engineering, AAV9-X1 exhibits high liver infectivity. AAV9-X1.1 was produced by introducing the CAP-B10 7-mer into VR-IV of AAV9-X1, improving production yield, but not decreasing liver transduction. To test whether the introduced CAP-B10 7-mer weakened PKD2 binding affinity, we performed BLI (Figures 3A and 3B). The K_d_ of AAV9-X1.1 for PKD2 was 19 μM, approximately three-fold weaker than AAV9-X1 (6 μM), suggesting that the diminished AAVR affinity of the CAP-B10 7-mer is transferable. Surprisingly, however, even with the VR-VIII loop modification, the K_d_ of AAV9-X1.1 was still comparable to that of wild-type AAV9 (11 μM) and considerably stronger than that of CAP-B10 (K_d_ 61 μM). The K_d_ of AAV9-X1 was not significantly different from that of AAV9, indicating that the VR-VIII loop of AAV9-X1 does not disrupt AAVR-PKD2 binding.

To further understand how AAVR affinity is maintained in AAV9-X1.1 despite the CAP-B10 VR-IV 7-mer, we explored our cryo-EM structures of AAV9-X1.1 with and without AAVR-PKD2, building atomic models of AAV9-X1, AAV9-X1.1, and the AAV9-X1.1–PKD2 complex, at resolutions of 3.09 Å, 2.51 Å, and 2.18 Å, respectively (Figures 1D, 3C-3E, S6A-S6C). AAV9-X1 and X1.1 both contain the VR-VIII 7-mer insertion ^587^AQGNNTRSV^7’^ (we denote the amino acids between 588 and 589 as G1’, N2’, N3’, T4’, R5’, S6’, V7’), differing from PHP.eB and CAP-B10 (^587^DGTLAVPFK^7’^) (Figures 1B and 1C). To analyze structural differences, we superimposed AAV9-X1 and AAV9-X1.1 with CAP-B10 (Figures S6B and S6C). AAV9-X1 and AAV9-X1.1 exhibited the same VR-VIII geometries, while AAV9-X1.1 VR-IV adopted the same geometry as in CAP-B10, indicating that the CAP-B10 structural motif is transferable independently of VR-VIII sequence. Analysis of the AAV9-X1.1 VR-IV loop in the PKD2-complexed structure showed that the CAP-B10 7-mer in AAV9-X1.1 was similarly reoriented, diminishing the AAVR-PKD2 interaction site, consistent with our hypothesized mechanism for reduction of AAVR affinity (Figure 3D). Similar to CAP-B10, we observed a conformational change in AAV9-X1.1 when binding PKD2 where A454 in the VR-IV loop is angled away from PKD2 (Figures 2G and 3D). However, unlike the lysine-lever-controlled VR-VIII of CAP-B10 (Figure 1C, Figure S1A), the VR-VIII loop of AAV9-X1.1 adopts a different conformation in complex with AAVR-PKD2 (Figure 3E **left**). To accommodate an anteriorly-bound AAVR-PKD2, the VR-VIII of AAV9-X1.1 orients such that four flexible groups, ^2’^NNTR^5’^, composed of polar and positive residues, can potentially interact with a negatively-charged patch on a posteriorly-bound AAVR-PKD2 (Figure 3E **right**). We hypothesize that the greater flexibility of the VR-VIII loop, minimizing steric clashing and enabling potential interaction with the negative patch on a posteriorly-bound AAVR-PKD2, may explain mechanistically why the affinity of AAV9-X1.1 for AAVR is higher than that of CAP-B10. This high AAVR-PKD2 affinity of VR-VIII, along with AAV9-X1.1’s interaction with LRP6^45^, which is expressed in the liver^34,35,42–44^, likely mitigate the liver de-targeting effect introduced by the CAP-B10 VR-IV motif.

### AAVR-dependent liver de-targeting is independent of brain sequestration

**Figure 4:**
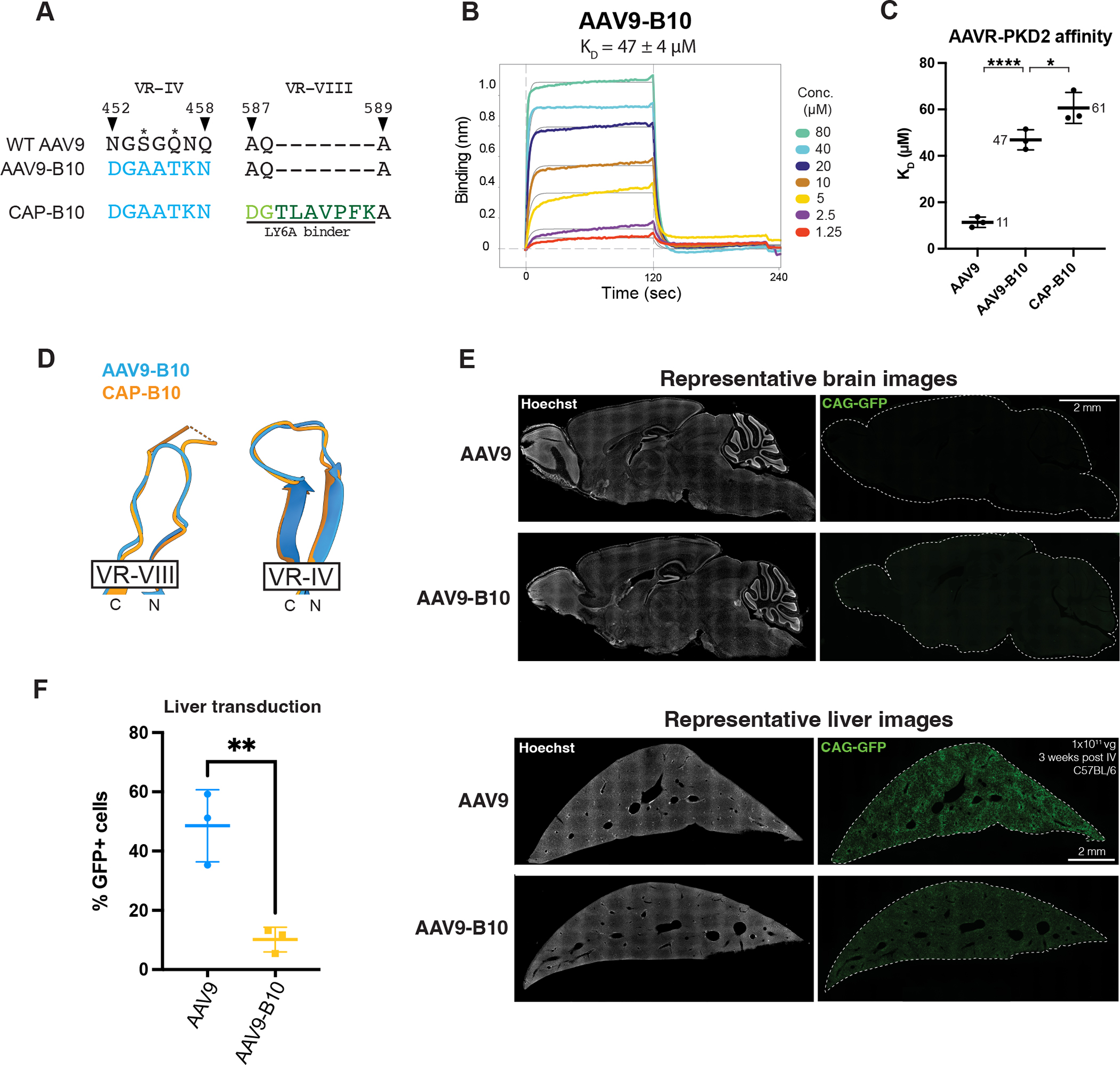
B10 structural motif can reduce liver transduction without brain sequestration. **(A)** Sequence alignment of VR-IV and VR-VIII in AAV9, AAV9-B10, and CAP-B10. AAV9-B10 contains the B10 7-mer substitution in VR-IV but lacks the PHP.eB 7-mer in VR-VIII, preventing interaction with LY6A. **(B)** Representative BLI sensorgram of AAV9-B10 binding to AAVRPKD2. The averaged Kd from triplicate measurements is shown above. **(C)** Comparison of the AAVR-PKD2 affinity values for AAV9, AAV9-B10, and CAP-B10. AAV9 and CAP-B10 data are repeated from Figures 2B and 2J for reference. **(D)** Cryo-EM structure of AAV9-B10, demonstrating that its VR-IV conformation is identical to that of CAP-B10. Additionally, AAV9’s native VR-VIII structure is not altered by the addition of the B10 structural motif in VR-IV. **(E)** Representative images of the brain and liver of mice following systemic delivery of AAV9 or AAV9-B10 packaging eGFP under the control of the CAG promoter. AAVs were retro-orbitally administered to the mice at a dose of 1×10^11^ vg per animal (n = 3 animals per capsid). eGFP expression was analyzed 3 weeks post-injection. Scale bars represent 2 mm. **(F)** Percentage of eGFP-expressing cells in the liver of mice receiving AAV9 or AAV9-B10. AAV9-B10 has markedly decreased liver transduction compared to AAV9 (**p < 0.01). Statistical significance was determined using ANOVA. *p < 0.05, **p < 0.01, and ****p < 0.0001.

AAVR is an essential host factor that mediates cell entry of most AAV serotypes^29,30^ and the significantly diminished interaction between liver de-targeted capsids and AAVR-PKD2 strengthens our initial hypothesis that reduced liver transduction is a result of weaker binding of AAVR-PKD2. PET imaging has shown that up to 35% of systemically-delivered PHP.eB is found in the brain in mice^46^, raising the possibility that liver de-targeting may alternatively reflect significant sequestration of AAVs from the bloodstream into the CNS. To test whether AAVR affinity modulation affects liver de-targeting for AAVs being developed and used to study and treat tissues outside the brain^32,33,36,37^, we incorporated the CAP-B10 7-mer into VR-IV of wild-type AAV9, creating AAV9-B10. This capsid cannot bind the receptor LY6A^47–50^ and therefore is not expected to accumulate in the brain to a significant level. BLI confirmed that AAV9-B10 has lower affinity (K_d_: 47 μM) than AAV9 for AAVR PKD2 (Figures 4A–4C). We then solved the structure of AAV9-B10 by single-particle cryo-EM at a resolution of 3.05 Å (Figures 4D, S6D-S6F). The resulting structure showed that the VR-IV loops of CAP-B10 and AAV9-B10 adopt similar geometries (Figure 4D). Additionally, the VR-VIII loop of AAV9-B10 was unaffected, retaining its wild-type AAV9 conformation. These results further demonstrate that the AAVR-PKD2 hindrance effect of VR-IV is highly transferable. To test the relative potency of AAV9 and AAV9-B10 in cells expressing AAVR but not LY6A, we conducted a cell infectivity assay in human embryonic kidney (HEK) 293T cells (Figure S7). AAV9-B10 showed less infectivity than AAV9, confirming reduced transduction from diminished AAVR affinity (Figure S7A).

To test this effect *in vivo*, AAV9 and AAV9-B10 packaging the reporter gene GFP were retro-orbitally injected into wild-type C57BL/6J mice (Figure 4E). After 3 weeks, brain and liver transduction levels were assessed. Transgene expression levels were quantified by counting GFP-positive cells, normalized to total cell counts determined by Hoechst staining. As expected, liver transduction of AAV9-B10 was significantly decreased compared to AAV9 (Figure 4F). Additionally, the GFP levels in the brain were comparable, and low, in both. These results suggest that the CAP-B10 7-mer substitution decreases transduction by reducing AAVR-PKD2 affinity in a manner that is independent of brain sequestration.

To assess whether reduced AAVR–PKD2 affinity also impacts the transduction of other organs besides the liver, we quantified transgene expression in skeletal muscle, heart, and pancreas–additional organs efficiently transduced by AAV9 (Figure S8). In the pancreas, AAV9-B10 transduced markedly fewer cells compared to AAV9, mirroring the reduction observed in the liver (Figure S8A). This indicates that the CAP-B10 7-mer insertion can similarly de-target AAV from the pancreas. In immunohistochemistry images of skeletal muscles (diaphragm and back muscle) and cardiac muscle, the differences in transduction levels between AAV9 and AAV9-B10 were less pronounced than in the liver and pancreas (Figures S8B, S8C). We confirmed this result by quantifying the relative enrichment of delivered GFP transgenes in each organ using droplet digital PCR (Figure S8D). In both skeletal muscles and heart, as expected from the immunohistochemistry, DNA enrichment levels were comparable or minorly decreased between AAV9 and AAV9-B10, while liver showed a stark decrease of delivered DNA from AAV9 to AAV9-B10. These results suggest that the tissue de-targeting effect conferred by the CAP-B10 structural motif varies across different organs.

### AAVR affinity of brain-enhanced AAVs governs de-targeting from the liver and CNS astrocytes

CAP-B10 is notable both for its liver de-targeting and for its enhanced specificity for neurons in the CNS^24^. To determine if both features arise from CAP-B10’s VR-IV 7-mer-mediated loss of affinity for AAVR, we next tested whether an intermediate AAVR affinity would yield an intermediate vector tropism. To test this hypothesis, we assessed three AAV9 derivatives with varying PKD2 affinities – PHP.eB (K_d_: 14 μM), CAP-B10 (K_d_: 61 μM), and eB.24 (K_d_: 24 μM) – *in vitro* and *in vivo*. As before, we first performed an *in vitro* infectivity assay in HEK293T cells (Figures S7B-S7D). As predicted, eB.24 displayed an intermediate level of infectivity, while PHP.eB and CAP-B10 showed the highest and lowest infectivity, respectively (Figures S7C and S7D). To see if the presence of an alternate receptor had any effect on transduction, we transfected the LY6A of C57BL/6J mice into HEK293T cells. Interestingly, all LY6A-dependent capsids demonstrated high infectivity in LY6A-expressing cells, with no significant differences between them, despite the substantial differences in AAVR PKD2 affinity among PHP.eB, eB.24, and CAP-B10 (Figure S7B). Taken together with the LY6A pulldown experiment (Figure S5D) these results suggest that reduced AAVR-PKD2 affinity, which reduces infection of the liver, can be compensated for by strong alternative receptors in the brain, such as LY6A.

**Figure 5:**
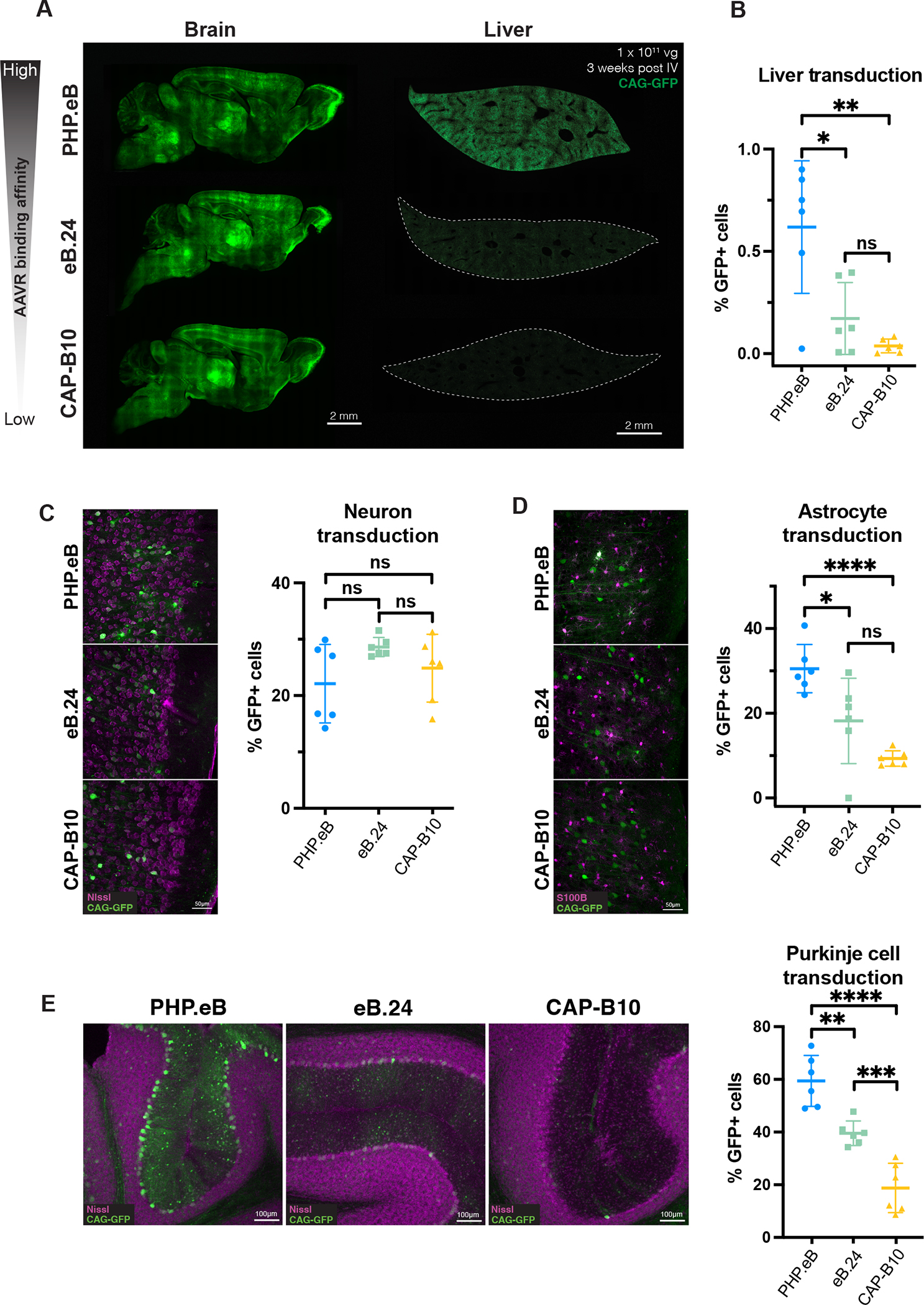
AAVR-PKD2 affinity modulates liver and brain tropism. **(A)** Representative fluorescence images showing eGFP expression in the brain and liver following retro-orbital delivery of PHP.eB, eB.24, or CAP-B10. AAVs packaging the fluorescent reporter eGFP under the ubiquitous CAG promoter were administered retro-orbitally at a dose of 1×10^11^ vg per mouse (n = 6 animals per capsid). eGFP expression was analyzed 3 weeks post-injection. The fluorescent reporter was used to quantify varying levels of brain and liver transduction. Scale bars represent 2 mm. **(B)** Quantification of eGFP-positive cells in the liver shows a correlation with the binding affinity for AAVR-PKD2. These findings demonstrate that liver tropism can be modulated by tuning AAVR-PKD2 affinity. **(C)** Representative images of eGFP-expressing cells co-stained with Nissl, a marker for neurons, to assess neuronal transduction in the cortex. The percentage of GFP+ neurons is consistent between PHP.eB, eB.24, and CAP-B10, suggesting that AAVR affinity does not significantly alter neuronal transduction levels. Scale bar represents 50 μm. **(D)** Percentage of eGFP-expressing cells co-localizing with S100B, indicating astrocyte transduction efficiency. Similar to the liver, astrocytic tropism decreases as AAVR-PKD2 affinity decreases, highlighting the role of AAVR binding in regulating astrocyte transduction. **(E)** Left: representative images of eGFP expression in the cerebellum delivered by PHP.eB, eB.24, or CAP-B10 where eGFP is in green and Nissl-stained neurons are in magenta. Right: percentage of eGFP-positive Purkinje cells in the cerebellum. As AAVR-PKD2 affinity decreases, the transduction level of Purkinje cells also decreases. Statistical significance was determined using a two-tailed unpaired t-test. *p < 0.05, **p < 0.01, ***p < 0.001, and ****p < 0.0001, ns not significant.

To test whether reduced AAVR affinity reduces liver transduction and biases brain tropism towards neurons, PHP.eB, eB.24, and CAP-B10 were retro-orbitally injected into wild-type C57BL/6J mice, with each capsid packaging GFP as a reporter gene. Liver transduction levels were measured by quantifying cells with GFP fluorescence, normalized to total cell count from Hoechst staining (Figures 5A and 5B). As expected, PHP.eB showed the highest level of liver transduction, and CAP-B10 the lowest. eB.24, consistent with its intermediate rank-order PKD2 affinity, exhibited intermediate liver transduction and significantly lower liver transduction than PHP.eB. These results are consistent with the hypothesis that attenuating the AAVR affinity of engineered AAVs can de-target capsids from the liver. We also measured GFP fluorescence in brain tissue to investigate the correlation between brain transduction efficiency and AAVR-PKD2 affinity. Consistent with previous reports^24^, qualitative analysis of brain slices revealed that CAP-B10 exhibited greater neuronal specificity compared to PHP.eB, which transduced both neuronal and astrocytic cells (Figure 5A). To further assess the brain cell-type specificity of these viruses, we performed immunohistochemistry using Nissl and S100B, markers for neuronal and astrocytic cells respectively (Figures 5C and 5D). PHP.eB and CAP-B10 achieved similar levels of neuronal transduction, with CAP-B10 exhibiting significantly lower astrocytic transduction compared to PHP.eB, reproducing previous results ^24^. Interestingly, eB.24 exhibited intermediate astrocytic specificity. When CAP-B10 was identified, another unique feature was observed: a large reduction in transduction of cerebellar Purkinje cells^24^. To see how well eB.24 emulates this behavior of CAP-B10, we quantified Purkinje cell transduction for PHP.eB, eB.24, and CAP-B10 (Figure 5E). As expected, CAP-B10 had much lower Purkinje cell transduction compared to PHP.eB. Interestingly, eB.24 exhibited a level of transduction intermediate between PHP.eB and CAP-B10. These observations suggest that AAVR affinity not only modifies liver targeting of AAVs but also selectively alters cell type tropism in brain-targeted engineered AAVs.

As demonstrated by our cryo-EM structure of eB.24 (Figures 2K, S4C-S4E), the overall VR-IV loop conformation is unchanged from that of PHP.eB, suggesting that the side chain interactions of the two mutated residues in eB.24 are sufficient to significantly redirect AAV away from the liver, brain astrocytes, and Purkinje cells in the cerebellum. CAP-B10 exhibits a further reduction in AAVR-PKD2 affinity, likely due to the additional structural rearrangement of VR-IV, which further de-targets liver, brain astrocytes, and cerebellar Purkinje cells. These results suggest that the effects of AAVR affinity on liver and brain cell-type tropism may operate along a gradient.

## Discussion

Directed evolution of AAV capsids has shown that vector transduction patterns across tissues and cell types can be dramatically altered in ways that are potentially advantageous. After intravenous administration, PHP.eB demonstrates broadly potent brain transduction in mice; the more recently identified CAP-B10 focuses that transduction to neurons of the CNS, largely de-targeting the liver (a source of serious adverse events in patients)^51–54^. Understanding the mechanisms behind these capsids’ potentially advantageous transduction properties may accelerate the creation of vectors better suited to human applications. While investigation of broadly potent CNS vectors, like PHP.eB, has yielded a host of new BBB transcytosis receptors, including a subset conserved in humans^45,47,55,56^, mechanisms of neuron specificity and liver de-targeting remain opaque.

**Figure 6:**
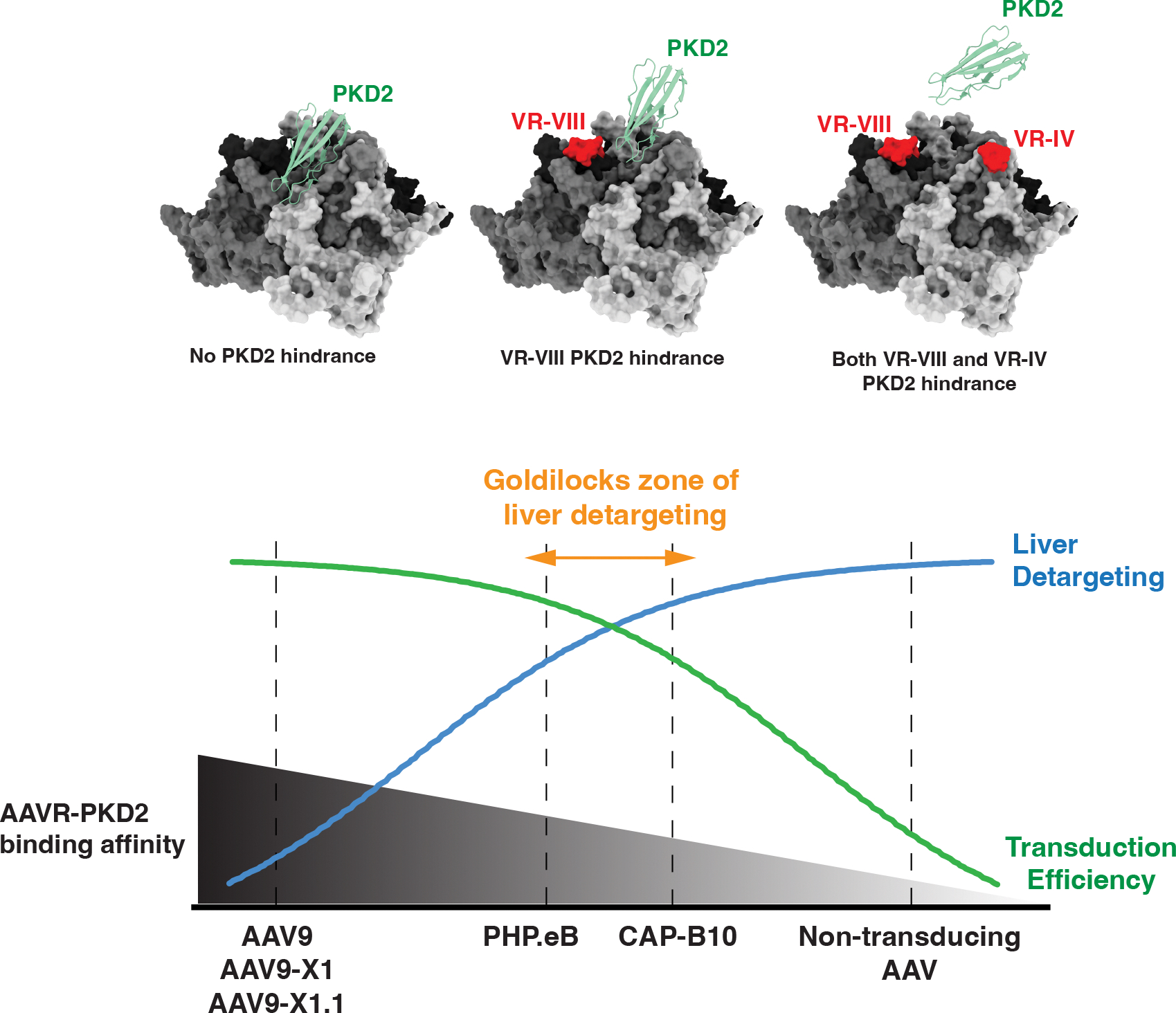
Engineering liver-de-targeted AAVs requires a balanced approach. While higher AAVR-PKD2 affinity generally enhances transduction across various organs, it also increases liver transduction. Conversely, AAVs that completely lose AAVR-PKD2 binding fail to transduce any cells. Additionally, even with reduced AAVR-PKD2 affinity, an AAV may still transduce the liver if it engages an alternative liver receptor, such as LRP6 for AAV9-X1.1. Therefore, designing liver-de-targeted AAVs requires optimizing AAVR-PKD2 affinity in the context of target-specific receptor interactions.

Here, based on detailed structural comparisons, binding characterizations, and *in vivo* observations, we reveal that CAP-B10 achieves liver de-targeting through reduced affinity for AAVR. Our analyses of previously reported (AAV9, CAP-B10, X1, X1.1) and newly developed (AAV9-B10, PHP.eB.24) capsids demonstrate that AAVR affinity operates as a dial governing both liver transduction and degree of neuronal specificity (Figure 6, Table 1). Our high-resolution structures allow us to further understand the molecular and structural mechanisms of AAVR interactions with engineered AAV capsids, providing a necessary foundation for future fine-tuning of receptor binding and, thus, *in vivo* tropism.

There have been efforts to engineer “canvas capsids^37^” with ablated basal infectivity as a starting point for the creation of vectors that might selectively target and/or de-target specific tissues ^27,38,57,58^. The CAP-B10 loop substitution in VR-IV offers a potential strategy for generating such canvas-like capsids with ablated liver infectivity by reducing AAVR-PKD2 affinity^59^. CAP-B10’s behavior appears to be explained by the gain of a LY6A interaction via PHP.eB 7-mer and a weakened AAVR interaction. However, AAVR knockout ablates PHP.eB transduction to near zero, and this effect cannot be rescued by LY6A^49^. CAP-B10’s requirement for weak-but-not-zero AAVR affinity may be explained by previous studies that suggest that AAVR might function not only as a cell surface receptor for AAV internalization but also as a dynamic intracellular trafficking factor^29,30^. If the internalization and trafficking roles of AAVR require different affinities, it is possible that CAP-B10 reduces AAVR-mediated off-target tissue cell internalization while still retaining AAVR-mediated intracellular trafficking in some cell types (Figure 6). Future work is needed to explore this and other possibilities. Our work highlights the importance of considering receptor binding by vectors not as an on/off switch but rather as a control dial, which can and should be tuned during AAV engineering. Detailed mechanisms of AAVR and LY6A function during cell transduction remain unknown, and designing an optimal receptor affinity balance will likely require a deeper understanding of the entire process from AAV uptake and trafficking to eventual nuclear import.

In our previous cryo-EM study^22^, we demonstrated that AAVR affinity can be controlled by altering the insertion length and geometry of VR-VIII. The present work expands this understanding of the AAVR interface to VR-IV^22^. Previously reported cryo-EM structures (PDB: 7WQO, 7WJX^39^) suggest a polar interface between PKD2 and the wild-type AAV9 VR-IV loop, including a hydrogen bond between Q456 of the wild-type AAV9 VR-IV loop and E418 from AAVR-PKD2. In our CAP-B10 structure, the substitution of Q456 with the more compact T456, the mutation of S454 to the hydrophobic A454, and the bending of the overall loop conformation away from PKD2, disrupts this polar interface. These changes correlate with a loss of AAVR-PKD2 affinity which is confirmed by BLI, suggesting that this polar interface contributes to a stable AAV-AAVR interaction.

We engineered a new capsid, eB.24, built on the PHP.eB backbone with two point mutations, S454A and Q456T, in the VR-IV loop. Unlike PHP.eB, this rationally designed capsid exhibited a PKD2 affinity both significantly weaker than AAV9 and significantly stronger than CAP-B10. Our cryo-EM structure of eB.24 showed that the overall loop shape of AAV9 VR-IV was not altered by the two mutations. This suggests that CAP-B10’s altered affinity for AAVR-PKD2 results from a combination of a loss of VR-IV side chain interactions, VR-VIII steric hindrance, and altered VR-IV loop conformation. Further work will be needed to fully understand the thermodynamic landscape of this interface between the AAV VR-IV loop and AAVR.

While the PKD2 affinity of eB.24 was not statistically different from that of PHP.eB, its affinity ranks between PHP.eB and CAP-B10. A similar trend was seen for PHP.eB, eB.24, and CAP-B10 in their liver, brain astrocyte, and cerebellar Purkinje cell transduction. This highlights how modest changes in PKD2 binding may induce substantial differences in vector tissue and cell type tropism *in vivo*. Additionally, we observed that PHP.eB, eB.24, and CAP-B10 show no difference in neuron transduction, suggesting a robustness to AAVR affinity modulation that may result from variations in receptor expression, warranting future study. These observations may explain a recent surprising result from pigtail macaques, where PHP.eB, whose BBB receptor LY6A is not present in non-human primates, exhibited more neuronal tropism than AAV9 following intracerebroventricular administration^60^.

Importantly, the effects of AAVR affinity modulation on liver transduction are not limited to brain-targeting AAVs. AAV9-B10, with its unaltered AAV9 VR-VIII and substituted CAP-B10 VR-IV loop, exhibited significantly lower AAVR affinity and liver transduction in mice than AAV9, and no increase in CNS transduction^21,24^. This suggests that diverse AAV capsids engineered to positively target non-CNS tissues may be similarly engineered to de-target the liver via AAVR affinity modulation, thereby improving their safety profiles.

Modulating AAVR affinity to reduce tissue transduction is not restricted to the liver, as we observed that AAV9-B10 transduction is also reduced in the pancreas relative to AAV9. This finding is consistent with our prior studies indicating that CAP-B10 shows reduced transduction compared to PHP.eB in various organs, including pancreas^24^. However, AAV9-B10 transgene expression in cardiac and skeletal (erector spinae, diaphragm) muscle was comparable to that of AAV9. This suggests that the B10 structural motif selectively de-targets specific organs. Muscle and heart are strongly transduced by AAV9 and are important targets for potential AAV-based gene therapies. It was previously reported that the introduction of a few point mutations into wild-type AAV9 leads to de-targeting from the liver without altering the transduction of heart and muscle^38^. While the molecular mechanism was not explored in that study, the location of the mutations (N498Y, N498I)^38^ is near the AAVR-PKD2 interaction site we characterize here. This leads us to believe that these mutations may similarly disrupt PKD2 interaction. It remains unclear however, whether an AAV’s overall organ or cell type selectivity profile arises from AAVR-independent factors, organ-specific differences in AAVR mechanism, or combinations thereof. (For example, why should cerebellar Purkinje neurons be more sensitive to weakened AAVR affinity?) Future studies on how AAVR affinity affects internalization and trafficking across various organs and cell types are needed.

We also tested the binding of LY6A and D-galactose to PHP.eB, eB.24, and CAP-B10 to assess whether the VR-IV modification affects non-AAVR receptors. Overall, the interaction levels remained largely unchanged, except for a slight reduction in D-galactose binding observed with CAP-B10 compared to PHP.eB and eB.24. While VR-IV modification is quite distant from the well-established D-galactose binding site on the 2-fold wall of the AAV surface ^26,27,61^, it is possible that the more polar residues in the protruding VR-IV region of PHP.eB may facilitate a non-specific polar interaction with D-galactose that is lost in CAP-B10. However, the monosaccharide D-galactose beads we used to assay binding do not fully mimic the glycoprotein environment of D-galactose on the cell surface, so further investigation is needed to determine how binding is affected *in vivo*.

Previous studies have identified VR-I engineering as an effective strategy for liver de-targeting and our structural analyses align with earlier work showing that the VR-I region is in close proximity to AAVR-PKD2^57,62,63^. Mapping these previously-identified mutations onto our AAV-PKD2 complex structures, we think it likely that they work by destabilizing the interaction between AAVR-PKD2 and VR-I. Notably, VR-I is also a well-characterized D-galactose binding site, and mutations are known to abolish the transduction capacity of AAV9^27,62^. Therefore, VR-I modification may represent another potential liver de-targeting strategy, through the combined disruption of AAVR and D-galactose interactions.

Cryo-EM structures of AAVR in complex with non-AAV9 serotypes, including AAV1, AAV2, and AAV5, have been determined previously^64–67^. Among these, AAV1 and AAV2 exhibit AAVR binding patterns similar to AAV9, with PKD2 engaging the VR-I, VR-IV, and VR-VIII regions. This structural homology suggests that liver de-targeting strategies based on reducing VR-IV and VR-VIII (and potentially VR-I) receptor interactions may be broadly applicable across gene delivery vectors based on multiple AAV serotypes.

Our analysis of a separate family of engineered capsids, AAV9-X1 and AAV9-X1.1, shows that the effect of diminished AAVR affinity can be masked by the contributions of other receptors. AAV9-X1.1 exhibits weaker AAVR affinity than AAV9-X1, indicating that the B10 loop hinders AAVR binding in multiple capsid variants. However, AAV9-X1.1’s affinity was higher than CAP-B10’s. This is likely because, unlike for CAP-B10, no structural motifs in VR-VIII appear to disrupt AAVR binding and the VR-VIII of X1/X1.1 may additionally participate in engaging AAVR. AAV9-X1 also uses a different receptor, LRP6, which is enriched in both the brain and liver. Knockout of LRP6 in the liver revealed that this receptor markedly boosts AAV9-X1.1 liver transduction^45^. These observations suggest that the effect of B10’s VR-IV structural motif on AAVR binding, and thus liver de-targeting, may be counteracted by LRP6-mediated liver transduction.

Here we present a broad structural study of the molecular mechanism by which brain-enhanced engineered AAVs achieve liver de-targeting, providing key insights into their tropism and therapeutic potential. By solving seven cryo-EM structures—five distinct AAV and two AAV–AAVR-PKD2 complex structures—we are able to postulate a molecular mechanism underlying this process. Our results suggest that CAP-B10 achieves improved liver de-targeting by reducing AAVR affinity. *In vivo* testing demonstrated that AAVR affinity correlates with liver transduction levels in both CNS-targeting and non-CNS-targeting capsids, and our designed mutant eB.24 suggests that it is possible to modulate AAVR affinity through strategic mutations to achieve a desired liver transduction level. Unexpectedly, we found that AAVR affinity also correlates with vectors’ brain cell type tropism. Combining VR-IV and VR-VIII modifications may provide a promising strategy for the creation of blank canvas capsids that de-target selected tissues through tuned AAVR affinity while specifically targeting desired tissues through an engineered interaction with another receptor. Optimizing this strategy will require careful selection of new target receptors such as LY6A, which, unlike LRP6, is not enriched in the liver. While this study focused on rodent CNS-targeting capsids, the mechanistic insights gained should be broadly applicable for future capsid engineering across tissues and species, including humans.

## Materials and Methods

### Animals

All mouse experiments were approved by the California Institute of Technology Institutional Animal Care and Use Committee (IACUC). Adult (6–8 weeks old) C57BL/6J WT male mice were purchased from the Jackson Laboratory (000664).

### Viral production

AAVs were produced as previously described^68^. Briefly, HEK293T cells were cultured in 150-mm dishes at 37 °C with 5% CO_2_ until they reached 80%–90% confluency. HEK293T cells were triple-transfected with pHelper, capsid (AAV9, PHP.eB, CAP-B10, eB.24, AAV9-B10, AAV9-X1, AAV9-X1.1), and genome plasmid (eGFP reporter gene controlled by SCP1 promoter for AAV9-X1.1, and eGFP reporter gene controlled by CAG promoter for other else), using polyethylenimine (PEI Max^®^, Polysciences) solution. Media was collected 72 hr after transfection, and media and cells were collected at 120 hr. AAVs were purified from cells and media through an iodixanol gradient. Purified AAVs were titered using droplet digital PCR (ddPCR, Bio-Rad).

### Protein preparation

For cryo-EM and pull-down assays, the PKD2 domain of AAVR (AA 401–498) was expressed in BL21 (DE3)-RIPL *E. coli* as a fusion protein carrying Myc and 6×His tags. Following cell lysis, insoluble material was removed by centrifugation at 20,000 ×g for 1 hour, and the resulting supernatant was loaded onto an Ni-NTA affinity purification column (Qiagen). The column was pre-equilibrated with 50 mM Tris-HCl (pH 8.0), 100 mM NaCl, and 20 mM imidazole, then washed sequentially with 20 mM Tris-HCl (pH 8.0), 20 mM imidazole, and NaCl gradients (500, 1,000, 500, and 150 mM) to remove non-specifically bound proteins. Elution was performed using 20 mM Tris-HCl (pH 8.0) and 100 mM NaCl, applying an imidazole step gradient (50, 100, 150, and 250 mM), with the 250 mM imidazole fractions collected. PKD2 monomer was separated using Superdex 200 size-exclusion chromatography column (Cytiva) for further experimentation.

For biolayer interferometry, the PKD2 domain (AA 401–498) was expressed in BL21 (DE3)-RIPL *E. coli* as a fusion protein carrying maltose binding protein (MBP) and 6×His tags. Following cell lysis, insoluble material was removed by centrifugation at 20,000 ×g for 1 hour, and the resulting supernatant was loaded onto an amylose resin (New England Biolabs). The column was pre-equilibrated with 50 mM Tris-HCl (pH 8.0), and elution was performed using 50 mM Tris-HCl (pH 8.0) and 10 mM maltose. PKD2 monomer was separated using a Superdex 200 size-exclusion chromatography column (Cytiva) for further experimentation.

For LY6A (AA 1–109) derived from C57BL/6 mice, a construct carrying Fc (human IgG1), Myc, and 6×His tags was transiently transfected into HEK293T cells at 80%–90% confluency using PEI. Conditioned media containing secreted LY6A with triple tags (Fc-Myc-6×His) were harvested 120 hours post-transfection, filtered through a 0.22-μm PES vacuum filter (Sigma-Millipore), and clarified. Ni-NTA resin (Qiagen) was used to capture His-Fc-LY6A, which was subsequently eluted with 100 mM NaCl, 50 mM Tris-HCl (pH 8.0), and 150 mM imidazole. The eluted protein was concentrated using a 10K Amicon concentrator (Sigma-Millipore) before downstream applications.

### Cryo-EM sample preparation and collection

3.5 μL of concentrated AAV (1×10^12^ vg/mL - 1×10^13^ vg/mL) was pipetted onto Quantifoil R 1.2/1.3 300 mesh Cu grids that had been glow discharged (Pelco EasiGlow, 10 mA, 1 min). Grids were then plunge-frozen using a Mark IV Vitrobot (FEI, now Thermo Fisher) (23 °C, 100% humidity, blot force 3, blot time 5 s). Grids were clipped and loaded onto either a 300 kV Titan Krios microscope (Thermo Fisher) equipped with a K3 6k × 4k direct electron detector (Gatan) or a 200 kV Talos Arctica microscope (Thermo Fisher) equipped with a K3 6k × 4k direct electron detector (Gatan). Micrographs were collected using SerialEM software^69^ with pixel sizes of 0.42 Å or 0.85 Å (Arctica and Krios, respectively) and a defocus range of 1.5–4 μm.

### Cryo-EM processing

Processing steps are outlined in Figure S3. In brief, raw movies were binned by 2 and gain and motion corrected in CryoSPARC(v4.1)^70^. CTF estimation was performed in CryoSPARC. Initial particle picks were generated with blob picker using a maximum diameter of 300 Å and a minimum diameter of 240 Å. Particles were inspected & filtered to eliminate obvious bad picks. The remaining picks were then extracted from micrographs with 2x binning. 2D classification with 100 classes was then used to exclude remaining junk particles. Ab-initio with one class was then run with C1 symmetry. Afterwards, a homogeneous refinement enforcing I1 symmetry was run, aligning most particles at a lower resolution. These particles were then re-extracted unbinned and used for another one class ab-initio with C1 symmetry. The results were fed into a homogeneous refinement with global CTF correction and enforced I1 symmetry. Finally, a non-uniform refinement was run and this map was used for post processing and subsequent model building in Phenix and COOT^40,41,71^.

A model of the VP1 capsid protein was built using AAV9 as a template (PDB: 3UX1^20^). This was fitted into the I1 capsid map using ChimeraX^72^. This was then fed into COOT^71^ and 7-mer insertions and/or substitutions were added or edited and manually fit into the density. The model and map were then iteratively refined in Phenix and manually tuned in COOT for several iterations before a final model was determined (Table S1). Analysis of models was done in ChimeraX. Buried surface area of the VR-IV (residues 450–460) was calculated using native ChimeraX commands based on the atomic model.

### Biolayer interferometry

The equilibrium dissociation constant (K_d_) values were determined using Blitz (formerly ForteBio, now Sartorius). A total of 4 μL of 0.2 mg/mL CaptureSelect^™^ biotin-conjugated AAV9 nanobody (Thermo Fisher^73^) was loaded onto an Octet^®^ SAX Streptavidin-coated biosensor (Sartorius) for 120 s. The biosensor was then incubated in 400 μL of running buffer (Dulbecco’s phosphate-buffered saline [DPBS, Gibco^™^], 1% BSA, 0.02% Tween-20) to wash out residual nanobody. Next, 4 μL of AAV at 5×10^12^ vg/ml was loaded onto the biosensor to form an AAV biolayer. After a 30 s washing step in 400 μL of running buffer, the biosensor was incubated with 4 μL of PKD2 for 120 s (association), followed by incubation in 400 μL of running buffer for 120 s (dissociation). After each cycle, the biosensor surface was regenerated by incubating it in 400 μL of 10 mM glycine pH 2.0 to remove bound AAV, and the AAV layer was reloaded at the beginning of each new cycle as described above. Sensorgrams were collected at least 6 times in the range of PKD2 concentration between 1.25 – 80 μM. Sensorgrams were analyzed using BLItz Pro software (formerly ForteBio, now Sartorius). K_d_ was measured from 3 experiments and averaged.

Statistical significance of differences in all measured K_d_ values was determined using ANOVA in GraphPad Prism (v10.3.1). For all statistical analysis, significance is represented as *p <0.05, **p < 0.01, ***p < 0.001, and ****p < 0.0001; non-significance, p > 0.05.

### Pull-down assays

Pull-down assays were performed as previously described^22,45^. Briefly, for protein receptor pull-down assay, prey AAVs (0.05 pmol) were mixed with 6xHis-tagged bait, either purified AAVR-PKD2 (100–200 pmol) or His-Fc-LY6A (30–60 pmol) and 15 μL Ni-NTA resin in a DPBS buffer with 20 mM imidazole for 1 hr at 4 °C in a rotary shaker in agitation mode. The mixture was loaded onto a spin column and washed twice with 100 μL (10 column volumes total) DPBS buffer with 20 mM imidazole to remove unbound AAVs. Prey AAVs interacting with the 6xHis-tagged bait were eluted with 60 μL DPBS buffer containing 150 mM imidazole. The resulting eluate was electrophoresed by SDS-PAGE and analyzed by western blotting with anti-VP1/VP2/VP3 (Arp, cat# 03–61058) and anti-6xHis (Abcam, ab18184) antibodies. Western blot images were analyzed using Image Lab (Bio-Rad).

For the D-galactose pull-down assay, D-galactose agarose resin (Thermo Fisher, cat# 20372) was prepared by mixing with Ni-NTA resin at ratios of 1:1, 1:2, and 0. Prey AAVs in DPBS were then mixed with D-galactose resin for 1 hr at 4 °C on a rotary shaker in agitation mode. Resin was then collected in a spin column, and prey AAVs interacting with D-galactose were eluted with DPBS buffer containing 100 mM D-galactose. The resulting eluate was processed as described above for AAVR PKD2 pull-downs.

### Retro-orbital injection and tissue preparation

Mice were retro-orbitally injected with AAVs (AAV9, PHP.eB, CAP-B10, eB.24, AAV9-B10) carrying CAG-GFP transgene under isoflurane (3–5%, inhaled), followed by administration of 1–2 drops of 0.5% proparacaine to the corneal surface.

Three weeks post-injection, mice were euthanized via intraperitoneal injection of 100 mg/kg euthasol (pentobarbital sodium and phenytoin sodium solution, Virbac AH). Animals were then transcardially perfused with 30 mL of ice-cold heparinized 0.1M PBS pH 7.4, followed by an equal volume of ice-cold 4% paraformaldehyde (PFA) in 0.1M PBS. Tissues were post-fixed in 4% PFA overnight at 4°C, rinsed, and preserved in 0.1M PBS with 0.05% sodium azide at 4 °C. Brain, liver, back muscle (erector spinae), and heart tissues were embedded in O.C.T. Compound and flash-frozen using a dry ice-ethanol bath. Brain and liver tissues were sectioned at 80 μm thickness, and heart and back muscle (erector spinae) tissues were sectioned at 50 μm thickness using a cryostat (Leica Biosystems) and stored in 1x PBS at 4 °C until further processing. The diaphragm was left intact for whole mount histology.

### Immunohistochemistry

Immunohistochemistry was performed on free-floating tissue sections in 1x PBS. For brain and liver tissues, sections were blocked with BlockAid Blocking Solution (ThermoFisher, B10710) containing 0.1% Triton X-100 (Sigma-Aldrich, #93443). This blocking buffer was also used for diluting primary and secondary antibodies. Tissue sections were incubated with primary antibodies overnight at 4 °C, followed by three 10-minute washes in 1x PBS with 0.1% Triton X-100. Subsequently, tissue sections were incubated with secondary antibodies for 2 hours at room temperature and washed three times for 10 min each with 1x PBS with 0.1% Triton X-100. For nuclear staining, tissue sections were incubated with 1/10000 Hoechst 33342 (ThermoFisher, H3570) in 1x PBS for 10 min, followed by three 10-min washes in PBS. For segmentation of neuronal cells, tissue sections were Nissl stained using 1/400 NeuroTrace 640/660 (ThermoFisher, N21483) in 1x PBS. After staining, tissues were washed twice for 1 hr each at room temperature, followed by one overnight wash at 4 °C in 1x PBS with 0.1% Triton X-100. After staining, sections were dried on slides, and Prolong Diamond Antifade Mountant (ThermoFisher, P36965) was applied to mount coverslips.

For cardiac and skeletal muscles, 1x PBS buffer was used for diluting primary and secondary antibodies. Whole organs and/or tissue sections were incubated with primary antibody overnight at 4 °C, followed by three 10-minute washes in 1x PBS with 0.1% Triton X-100. Subsequently, tissue sections were incubated with secondary antibodies for 2 hours at room temperature and washed three times for 10 min each with 1x PBS with 0.1% Triton X-100.

The following antibodies were used in this study: rabbit anti-S100B (1/500, Abcam, ab52642), F(ab’)2 donkey anti-rabbit Alexa Fluor 647 (1/1000, Jackson ImmunoResearch, #711–606-152), chicken anti-eGFP (1/1000, Antibodiesinc, #GFP-1020), F(ab’)2 donkey anti-chicken Alexa Fluor 647 (1/1000, Jackson ImmunoResearch, #703–606-155).

### imaging and analysis

#### Brain and liver:

For overview images of mouse brain and liver sections, a Keyence BZ-X710 epifluorescence microscope was used, with a 10x, 0.45 NA air objective.

For imaging fields of view for brain and liver quantification, a Zeiss LSM 880 with a 25x, 0.8 NA water immersion objective was used. Imaging settings were chosen to capture the full dynamic range of the signal without saturating pixels. When possible, laser power was adjusted before adjusting detector gain. Imaging settings were first optimized on control samples before imaging of experimental samples. Fields of view were chosen while imaging non-experimental channels (e.g. Hoechst or S100B). Three non-adjacent sections were imaged in the cortex, cerebellum, and liver to compensate for local heterogeneity in these tissues. For each section, three fields of view were taken to minimize heterogeneity within quantification.

For liver transduction quantification, cellular nuclei were segmented from the Hoechst channel in CellProfiler (v4.2.5; https://cellprofiler.org/)^74^. Segmented nuclei were then used as a mask on the GFP channel. The GFP intensity was quantified from the masked images. The images were then filtered into positive or negative cells, allowing for the percentage of GFP+ cells to be calculated.

Classification of cortical cells as neuronal GFP-positive or astrocytic GFP-positive was done using CellProfiler. Astrocytes were segmented using the S100B channel in CellProfiler, while neuronal cells were segmented from the Nissl channel using Cellpose (v3.0.7; https://www.cellpose.org/). Images were batch processed using napari (v0.4.19.post1; https://napari.org/stable/) and the serialcellpose plugin (v0.2.2; https://www.napari-hub.org/plugins/napari-serialcellpose). An Anaconda (v2.5.4; https://www.anaconda.com/) distribution of Python (v3.10.14; https://www.python.org/) was used. The GFP channel was segmented using the DAPI channel and then thresholded in CellProfiler. All thresholds were chosen based on distribution of mean GFP intensity in soma. In both S100B and Nissl samples the colocalization between GFP and the cell type marker was calculated in CellProfiler.

#### Pancreas, skeletal muscles, and heart:

For overview imaging of pancreas, a spinning disc confocal microscope (Dragonfly, Andor; Fusion for software control) coupled with an sCMOS camera (Zyla, Andor) was used. Images were acquired with ×10/×25 objectives (Leica).

For imaging fields of view for pancreas, skeletal muscles, and heart, a spinning disc confocal microscope (Dragonfly, Andor; Fusion for software control) coupled with an sCMOS camera (Zyla, Andor) was used. Images were acquired with ×10/×25 objectives (Leica). Area-normalized eGFP expression level was measured by Fiji^75^.

Statistical significance of all measured tropism was determined using a two-tailed unpaired t-test in GraphPad Prism(v10.3.1). For all statistical analysis, significance is represented as *p <0.05, **p < 0.01, ***p < 0.001, and ****p < 0.0001; non-significance, p > 0.05.

### Droplet digital PCR

The droplet digital PCR (ddPCR) protocol used in this study is available on protocols.io (https://doi.org/10.17504/protocols.io.8epv5r84dg1b/v1)^76^.

For the quantification of AAV genomes from bulk tissue, DNA was extracted using a Quick-DNA FFPE Miniprep kit (Zymo Research, cat# D3067). A detailed protocol is available from the manufacturer’s website (https://www.zymoresearch.com/products/quick-dna-ffpe-miniprep?srsltid=AfmBOorwblfGX4QjhXsSrAzzINgaK5JlsWznDtws1xHNVinj2FUY-bVg).

1 μg of extracted DNA was digested overnight at 37 °C using 20 U of SphI-HF and SpeI-HF (New England Biolabs, R3182 and R3133). The digested DNA was used undiluted (for back muscle, diaphragm, and heart samples) or diluted 1:5 (for liver samples), and 5 μL (for liver, diaphragm, and heart samples) or 10 μL (for back muscle samples) was loaded into a 25 μL PCR reaction (Bio-Rad, 1863024). 23 μL of the PCR reaction was used to generate droplets (Bio-Rad, 1863005) on a QX200 Droplet Generator (Bio-Rad). 45 μL of droplets were transferred to a PCR plate for PCR amplification. After amplification, droplets were analyzed on a QX200 Droplet Reader using QX Manager software (Bio-Rad, 12010213). EGFP and WPRE sequences were detected using double-quenched FAM- and HEX-labeled probe assays, respectively (Integrated DNA Technologies). Droplets positive for both sequences were counted as true positives and used to calculate the number of AAV genomes per ng of total input DNA. Primer and probe sequences used for ddPCR are listed in Table S3.

Statistical significance of all measured tropism was determined using a two-tailed unpaired t-test in GraphPad Prism(v10.3.1). For all statistical analysis, significance is represented as *p <0.05, **p < 0.01, ***p < 0.001, and ****p < 0.0001; non-significance, p > 0.05.

### Cell infectivity assay

Cell infectivity assays were performed as previously described^45,47^. Briefly, HEK293T cells (ATCC, CRL-3216) were maintained in Dulbecco’s Modified Eagle Medium (DMEM) supplemented with 5% fetal bovine serum (FBS), 1% nonessential amino acids (NEAA), and 100 U/mL penicillin-streptomycin, at 37 °C with humid air containing 5 % CO_2_. Cells were seeded in 6-well plates, and at 80% confluency, cells were transfected with 2.53 μg of plasmid DNA containing a LY6A expression sequence. Cells were then transferred to 96-well plates at 20% confluency in FluoroBrite^™^ DMEM supplemented with 0.5% FBS, 1% NEAA, 100 U/mL penicillin-streptomycin, 1× GlutaMAX, and 15 μM HEPES. Cells expressing LY6A were transduced with AAVs of interest at 1×10^9^ or 5×10^9^ vg per well in triplicate. 24 hours later, cells were imaged with a Keyence BZ-X700 microscope with a 4× objective. Images were quantified as previously described, using our custom Python-based image processing toolkit (https://github.com/GradinaruLab/in-vitro-transduction-assay.).

## Supplementary Material

1

## Figures and Tables

**Table 1:** AAVR-PKD2 binding affinity ranking, and *in vivo* tropism of capsids used in this study.

Capsid	AAV9 engineered sequences		AAVR-PKD2 affinity rank	K_d_ against AAVR-PKD2 (μM)	Systemic Liver transduction	Mouse CNS tropism	Engineered Receptor	Receptor Distribution		Ref.
	VR-IV AA 452–458	VR-VIII AA 587–590						Liver	CNS	
AAV9-X1	NGSGQNQ	AQ***GNNTRSV***AQ	1	6	+++	Endothelial	LRP6	O	O	^25^
AAV9 (wild-type)	NGSGQNQ	AQ-------AQ	2	11	+++	Weak	-			^4^
PHP.eB	NGSGQNQ	**DGTLAVPFK**AQ	3	14	++	Neuronal & Astrocytic	LY6A	X	O	^23^
AAV9-X1.1	**DGAATKN**	AQ***GNNTRSV***AQ	4	19	+++	Endothelial	LRP6	O	O	^25^
eB.24	NG**A**G**T**NQ	**DGTLAVPFK**AQ	5	24	+	Neuronal & Less Astrocytic	LY6A	X	O	This study
AAV9-B10	**DGAATKN**	AQ-------AQ	6	47	++	Weak	-			This study
CAP-B10	**DGAATKN**	**DGTLAVPFK**AQ	7	61	Very low	Highly Neuronal	LY6A	X	O	^24^

**Bold** residues: CAP-B10 substitution

**Bold & italic** residues: LRP6 interacting motif

**Bold & underlined** residues: LY6A interacting motif

## Data Availability

Cryo-EM maps and atomic models have been deposited with the accession codes EMDB:71273 and PDB:9P4L (CAP-B10), EMDB:71278 and PDB:9P4Q (CAP-B10 - PKD2), EMDB:71277 and PDB:9P4P (AAV9-X1), EMDB:71279 and PDB:9P4R (AAV9-X1.1), EMDB:71275 and PDB:9P4N (AAV9-X1.1 - PKD2), EMDB:71274 and PDB:9P4M (AAV9-B10), and EMDB:71276 and PDB:9P4O (eB.24).
