# Supplementary material for: Structural basis of liver de-targeting and neuronal tropism of CNS-targeted AAV capsids
Source: Mol Ther. Author manuscript; Available in PMC 2026 Jun 16. (PMC13267735; doi:10.1016/j.ymthe.2026.03.030)
Supplement: 1 [file NIHMS2167070-supplement-1.pdf]

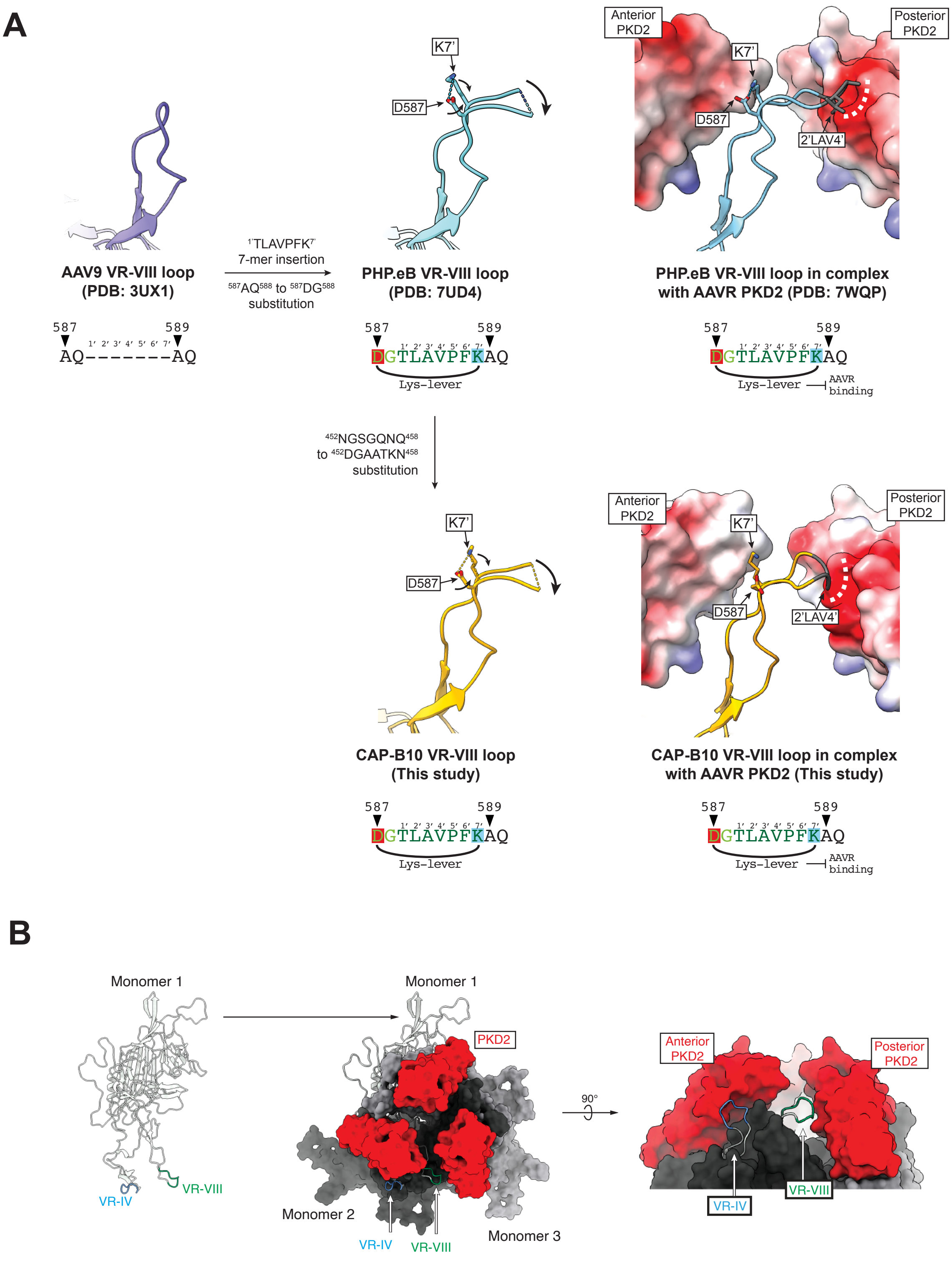

Figure S1: PHP.eB and CAP-B10 interaction with PKD2. (A) PHP.eB has a 7-mer insertion at VR-VIII ($^{1'}$TLAVPFK$^{7'}$) between AA 588 and 589, and two adjacent point mutations (^{587}AQ^{588} to ^{587}DG^{588}). In a previous study, we identified that the acidic residue D587 forms a hydrophilic interaction with the basic residue K7' from the 7-mer, which we termed the 'lysine-lever'. This interaction creates structural tension, causing VR-VIII to bend inward. The inwardly bent conformation introduces a steric clash with AAVR-PKD2, thereby reducing the binding affinity. This motif is conserved in CAP-B10, thereby interfering with AAVR-PKD2 binding. AAV9 (PDB: 3UX1[19]) is shown in purple. In PHP.eB alone (PDB: 7UD4[21]) and PHP.eB - PKD2 complex (PDB: 7WQP[38]), PHP.eB is shown in blue. In CAP-B10 alone (This study) and CAP-B10 - PKD2 complex (This study), CAP-B10 is shown in yellow (This study). PKD2 is presented with electrostatic mapping. **(B)** Illustration of how VR-IV and VR-VIII from a single AAV monomer interact with two PKD2 molecules. The three AAV monomers which compose the 3-fold face are shown in white, light gray, and dark gray. AAVR-PKD2 is red. VR-IV and VR-VIII are blue and green, respectively. VR-IV and VR-VIII are positioned between two neighboring monomers, allowing them to interact with both. The posterior PKD2 interacts with the protruding end of VR-VIII, including the PHP.eB 7-mer, while the anterior PKD2 interacts with VR-IV and VR-VIII.

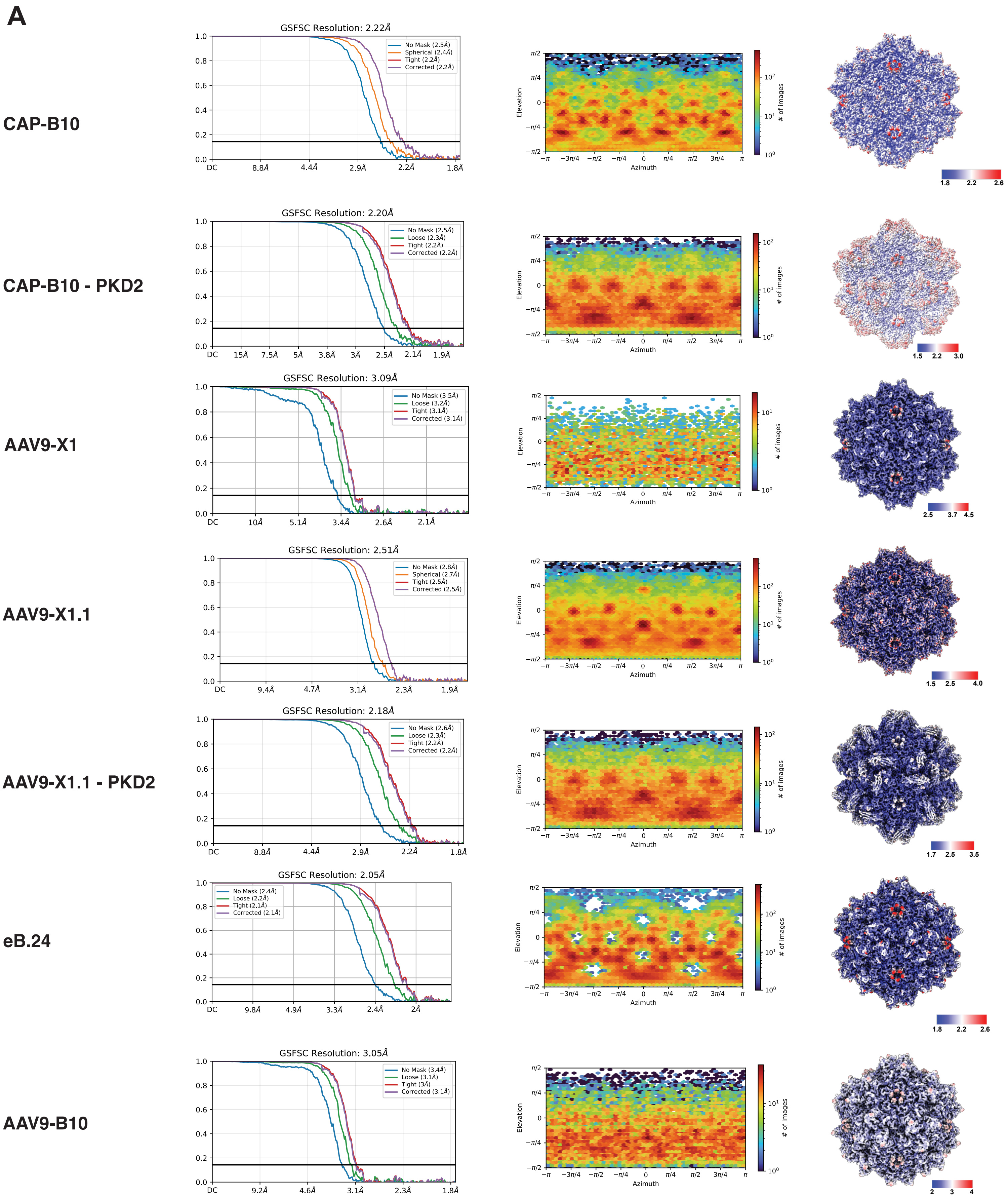
A
CAP-B10
GSFSC Resolution: 2.22Å
No Mask (2.5Å)
Spherical (2.4Å)
Tight (2.2Å)
Corrected (2.2Å)
CAP-B10 - PKD2
GSFSC Resolution: 2.20Å
No Mask (2.5Å)
Loose (2.3Å)
Tight (2.2Å)
Corrected (2.2Å)
AAV9-X1
GSFSC Resolution: 3.09Å
No Mask (3.5Å)
Loose (3.2Å)
Tight (3.1Å)
Corrected (3.1Å)
AAV9-X1.1
GSFSC Resolution: 2.51Å
No Mask (2.8Å)
Spherical (2.7Å)
Tight (2.5Å)
Corrected (2.5Å)
AAV9-X1.1 - PKD2
GSFSC Resolution: 2.18Å
No Mask (2.6Å)
Loose (2.3Å)
Tight (2.2Å)
Corrected (2.2Å)
eB.24
GSFSC Resolution: 2.05Å
No Mask (2.4Å)
Loose (2.2Å)
Tight (2.1Å)
Corrected (2.1Å)
AAV9-B10
GSFSC Resolution: 3.05Å
No Mask (3.4Å)
Loose (3.1Å)
Tight (3Å)
Corrected (3.1Å)
Elevation
Azimuth
# of images

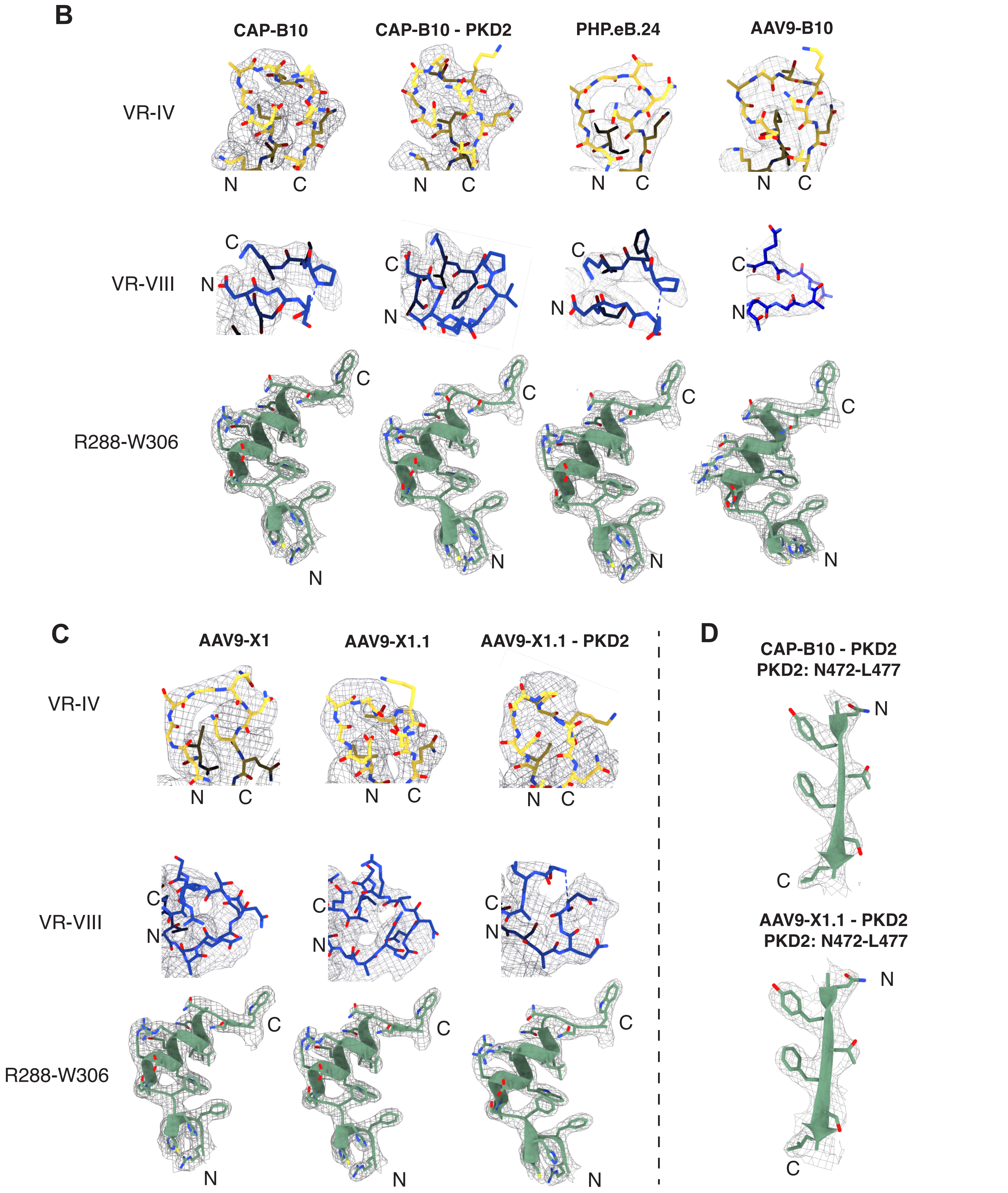

Figure S2: Resolution of all AAV structures determined in this study. (A) Gold standard FSC curves with indicated resolution at 0.143 cutoff (left), azimuth elevation plots (middle), and local resolution of each cryo-EM map (right). **(B, C)** Electron density maps of VR-IV (AA 450-456), VR-VIII (AA 587-589 with 7-mer insertion if it exists), and R288-W306 regions of CAP-B10, CAP-B10-PKD2 complex, PHP.eB.24, AAV9-B10 **(B)** or AAV9-X1, AAV9-X1.1, AAV9-X1.1-PKD2 complex **(C)** cryo-EM maps with associated atomic models. **(D)** Electron density maps of N472-L477 of PKD2 in CAP-B10 -PKD2 complex and AAV9-X1.1 - PKD2 complex structures with associated atomic models.

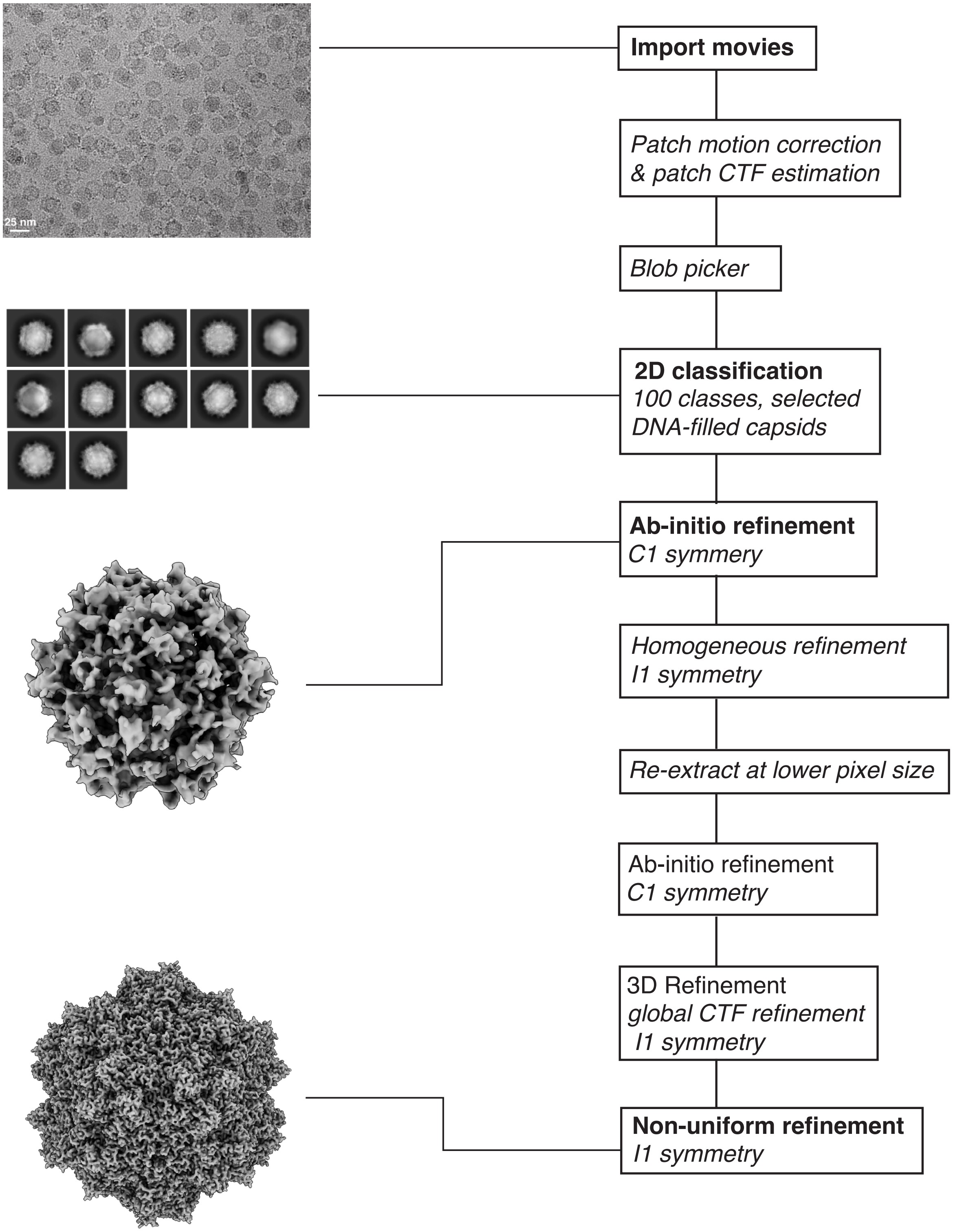

Figure S3: Cryo-EM single particle reconstruction processing pipeline. All cryo-EM structures in this study were processed using cryoSPARC (v4.1). Details of steps and parameters used are described in the Methods and Supplementary Table 1. Briefly, raw movies were binned by 2 and gain and motion corrected in CryoSPARC(v4.1)[63] CTF estimation was performed in CryoSPARC . Initial particle picks were generated with blob picker and were inspected to eliminate bad picks. 2D classification with 100 classes was then used to exclude remaining junk particles. Multiple rounds of refinement jobs in I1 symmetry were performed on good particles to construct the final map for model building. A model of the VP1 capsid protein was built based on AAV9 as a template (PDB: 3UX1[19]), using Phenix and COOT[39,40,64].

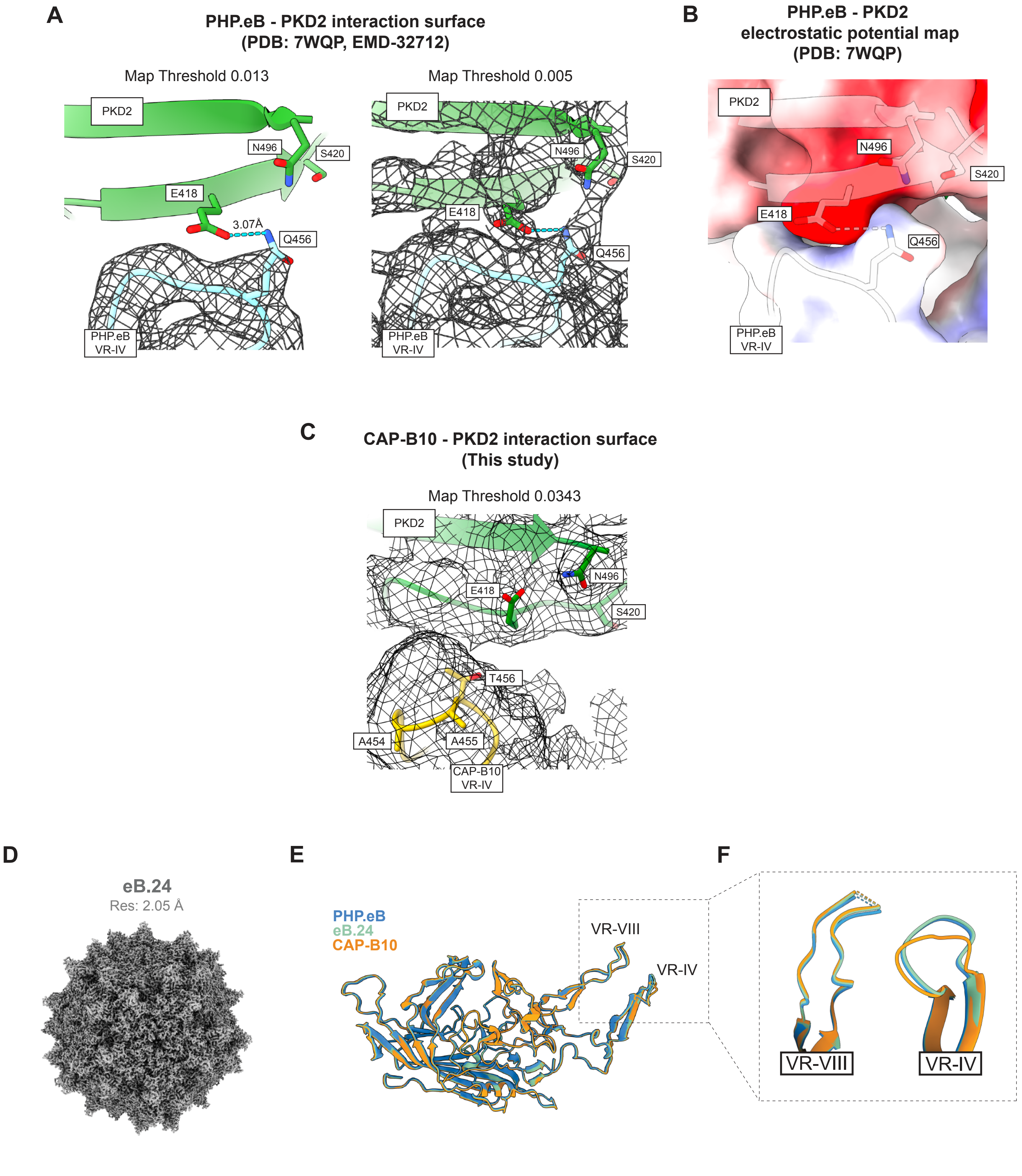

Figure S4: Supporting structure information for Figure 2. (A) Hydrogen bonding interaction between PKD2 and PHP.eB VR-IV (PDB: 7WQP, EMD-32712). The Q456 of PHP.eB might form a hydrogen bond with E418 of PKD2 at a distance of 3.07 Å. **(B)** Electrostatic potential of interaction face shown in **A**. Highly negative PKD2 surface is engaging with polar-and-mild-positive surface of PHP.eB VR-IV loop. **(C)** Electron density of CAP-B10 and PKD2 complex. Both atomic model and electron density map are from this study. **(D–F)** Cryo-EM structure of our designed AAV, eB.24 **(D)**, and a cryo-EM-based atomic model of eB.24 overlaid with PHP.eB (PDB: 7UD4) and CAP-B10 (this study) **(E, F)**. The backbone structures of VR-IV are identical in eB.24 and PHP.eB, whereas CAP-B10 differs, indicating that the S454A and Q456T mutations are insufficient to alter the overall loop structure. PHP.eB, eB.24, and CAP-B10 all share an identical backbone structure of VR-VIII.

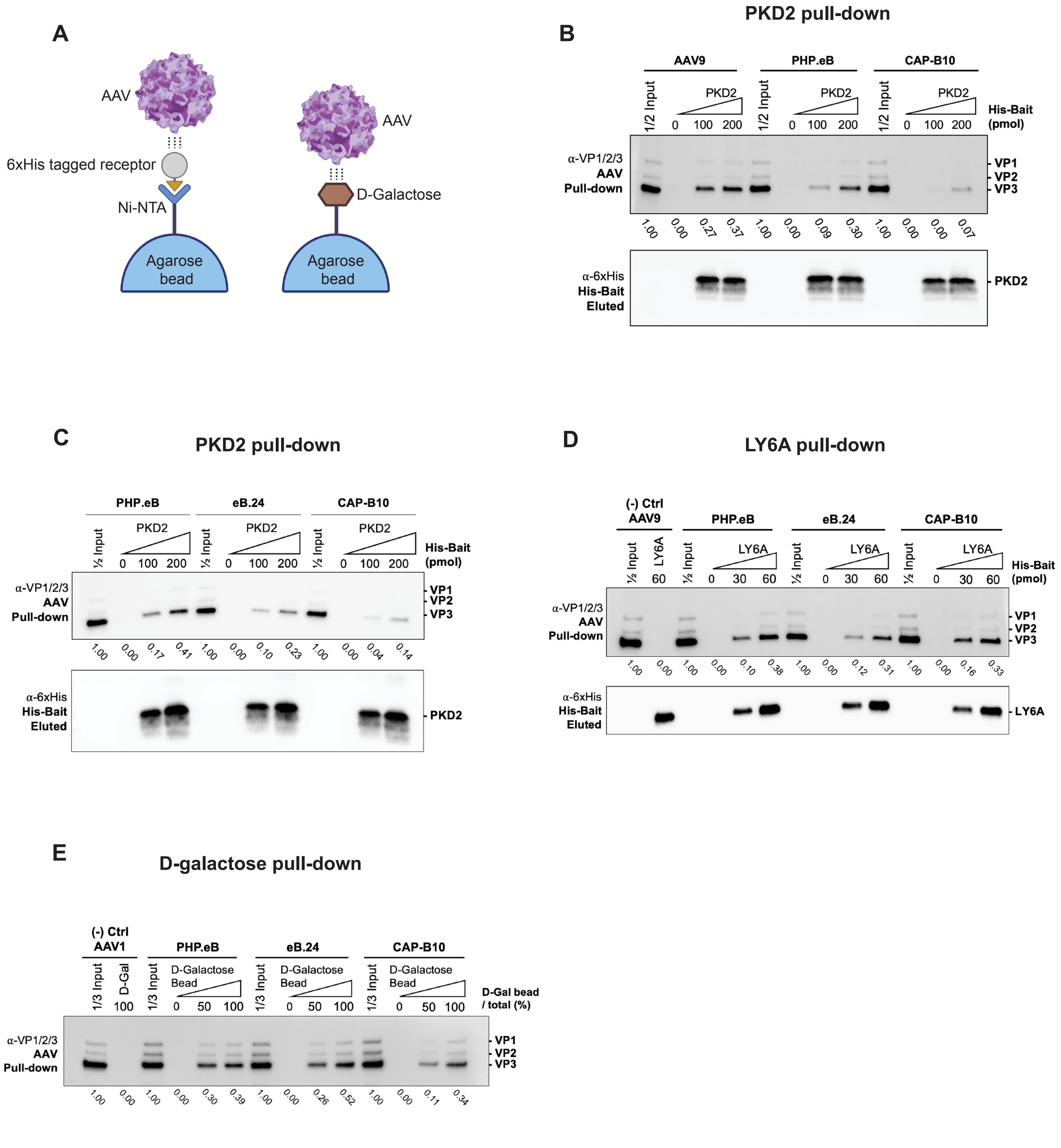

Figure S5: AAV-receptor pull-down assay. (A) Schematic of pull-down assay, using receptor as bait and AAV as prey. For protein receptors, receptors tagged with 6xHis were immobilized on agarose beads, and the amount of AAV captured by the receptor was analyzed using Western blot. For D-galactose receptor, D-galactose pre-loaded agarose beads were used. To prevent signal saturation, either ½ or ⅓ of the input was loaded. The amount of AAV pulled down was normalized to the input and is represented below each lane. **(B)** Pull-down of AAV9, PHP.eB, and CAP-B10 by PKD2. CAP-B10 was pulled down the least, consistent with the relative ranking of binding affinities measured by BLI. **(C-E)** Pull-down of PHP.eB, eB.24, and CAP-B10 by PKD2, LY6A, or D-galactose. **(C)** The degree of VR-IV modification of the capsids showed an inverse correlation with PKD2 binding strength. **(D,E)** LY6A and D-galactose binding did not show a similar trend, suggesting that VR-IV modifications specifically impact AAVR-PKD2 interaction.

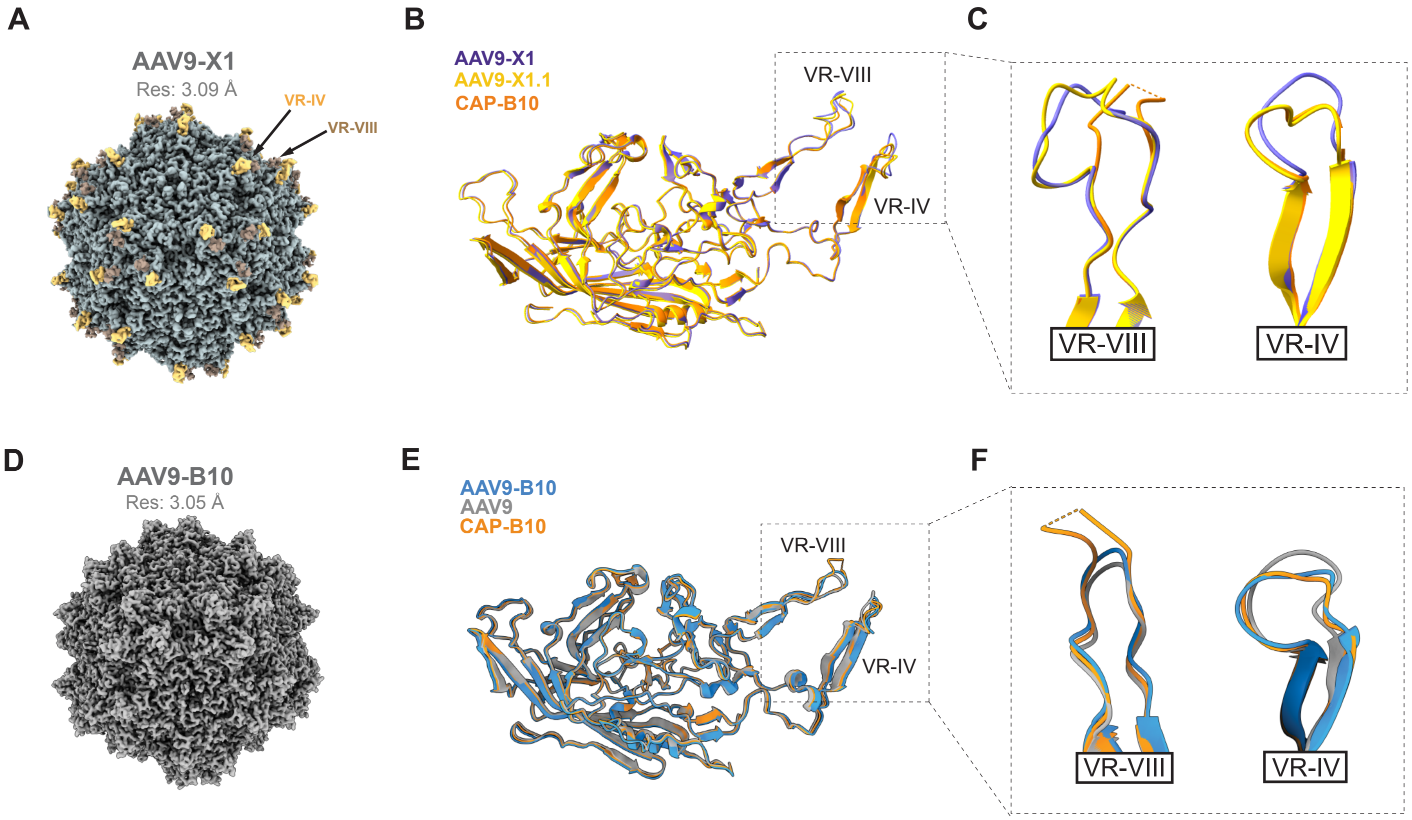

Figure S6: Supporting structure information for Figures 3 & 4. (A) Cryo-EM map of AAV9-X1. **(B, C)** Atomic model of AAV9-X1 (purple), showing whole monomer, VR-IV, and VR-VIII regions, superimposed on CAP-B10 (orange), AAV9-X1.1 (yellow) to highlight structural differences and similarities. The VR-VIII loop structure is similar between AAV-X1 and AAV9-X1.1. AAV9-X1 exhibits a distinct VR-IV structure compared to CAP-B10 and AAV9-X1.1. AAV9-X1.1 and CAP-B10 share an identical VR-IV loop structure. **(D)** Cryo-EM reconstruction of AAV9-B10 structure. **(E, F)** Atomic model of AAV9-B10, showing the full capsid, VR-IV, and VR-VIII regions, built based on the cryo-EM structure (blue). These models are superimposed with CAP-B10 (orange) and AAV9 (gray) to illustrate structural differences and similarities. AAV9-B10 and CAP-B10 share an identical VR-IV loop structure.

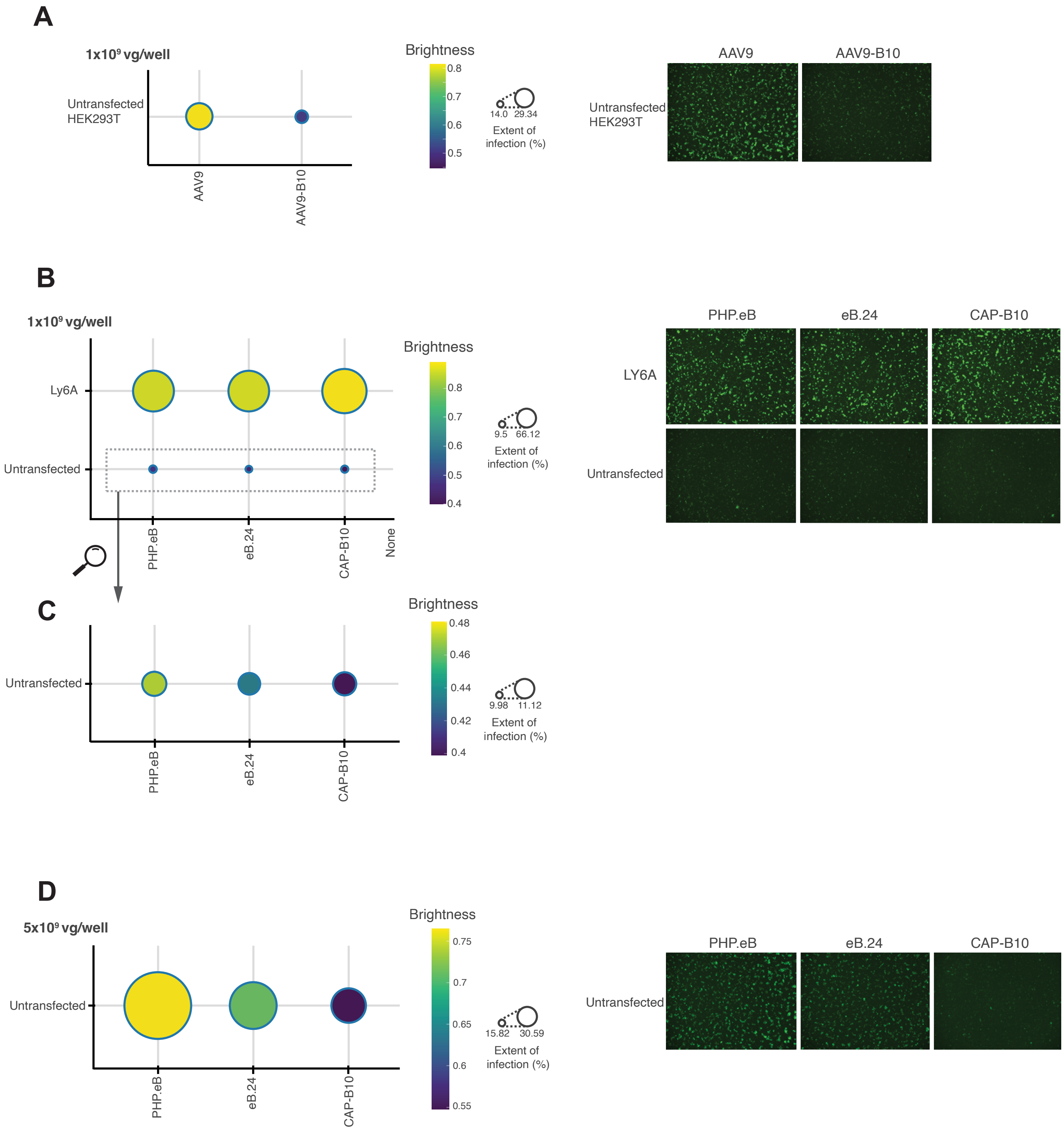

Figure S7: Cell infectivity assays of various capsids in HEK293T cells. Each panel presents the transduction efficiencies of capsids in HEK293T cells either untransfected or transfected with a construct to express LY6A on their membranes. Color indicates extent of infection and size indicates total brightness per signal area. Data were quantified from triplicate experiments. Representative fluorescence images of HEK293T cells expressing eGFP delivered by each capsid are shown on the right. **(A)** AAV9, AAV9-B10 were tested at a dosage of $1{\times}10^9$ vg / well. **(B)** PHP.eB, eB.24, and CAP-B10 were tested at a dosage of $1{\times}10^9$ vg / well in both untransfected or LY6A-expressing HEK293T cells. **(C)** Rescaled comparison of PHP.eB, eB.24, and CAP-B10 in untransfected HEK293T cells (lacking LY6A), revealing a weak inverse correlation between PKD2 binding affinity and transduction efficiency, though basal transduction levels were low. **(D)** Cell infectivity of PHP.eB, eB.24, and CAP-B10 at a higher dosage of $5{\times}10^9$ vg per well. The inverse correlation between PKD2 binding affinity and cell transduction potency became more apparent. However, this trend was not observed in LY6A-expressing cells, suggesting that alternative receptors can override the effects of reduced PKD2 binding.

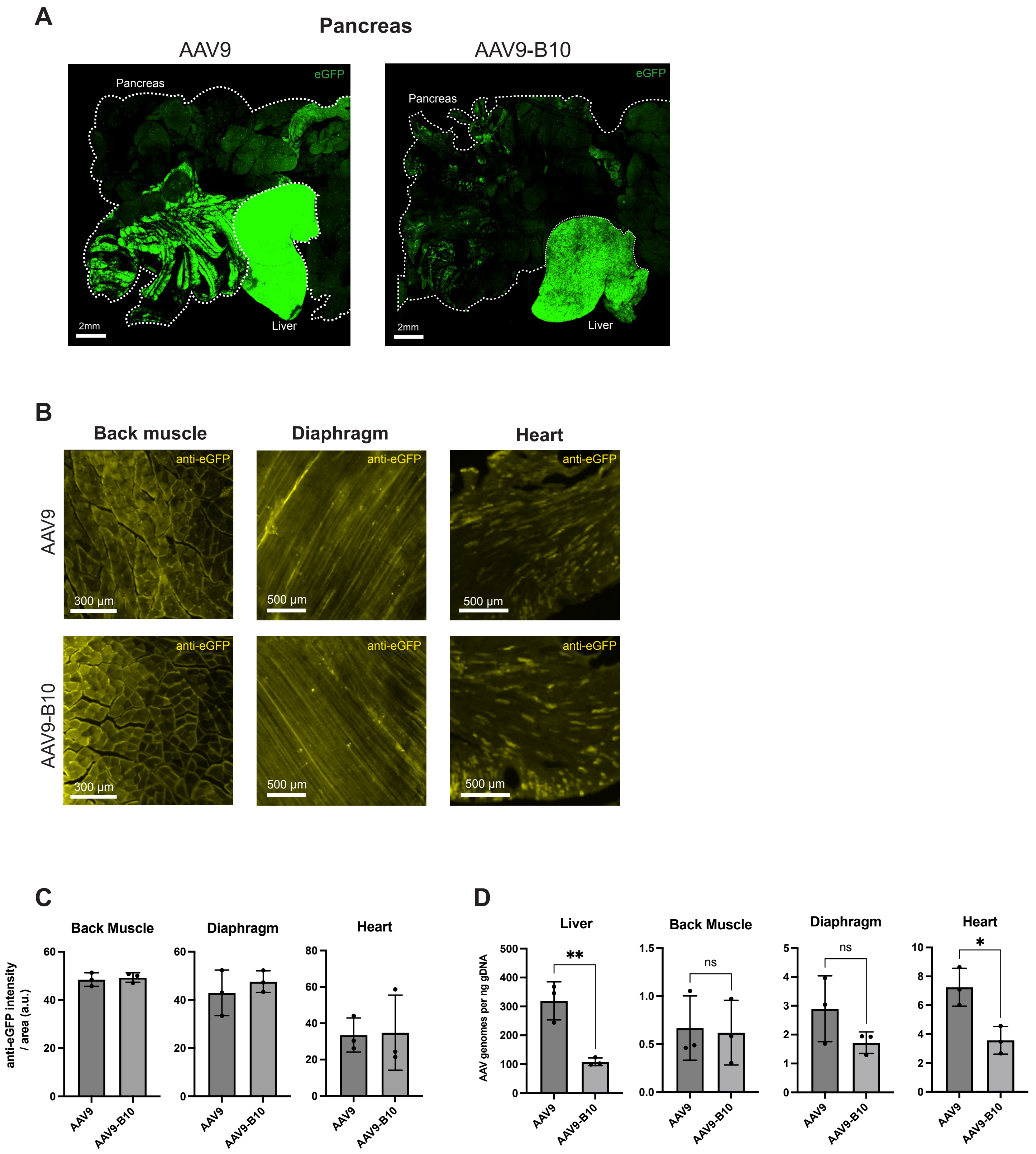

Figure S8: eGFP expressions in pancreas, skeletal muscles, and heart. (A) Head side of the pancreas, with liver as a positive control. **(B)** Representative anti-eGFP immunohistochemistry images of back muscle (erector spinae), diaphragm, and cardiac muscle of heart. **(C)** Area-normalized anti-eGFP intensity of each tissue. **(D)** Quantification of delivered AAV genome enrichment by droplet digital PCR. Statistical significance was determined using unpaired t-tests. Bars are mean ± s.e.m. p < 0.05, **p < 0.01, ns not significant.

Table S1. Cryo-EM data collection and validation statistics

	CAP-B10	CAP-B10 PKD2 complex	AAV9-X1	AAV9-X1.1	AAV9-X1.1 PKD2 complex	eB.24	AAV9-B10
EMDB	71273	71278	71277	71279	71275	71276	71274
PDB	9P4L	9P4Q	9P4P	9P4R	9P4N	9P4O	9P4M
Magnification	x105,000	x105,000	x105,000	x105,000	x105,000	x105,000	X45,000
Voltage(kV)	300	300	300	300	300	300	200
Electron exposure(e^{-}/Å^{2})	60	60	60	60	60	60	60
Defocus range(μm)	-1 to -3	-1 to -3	-1.5 to -3	-1.5 to -3	-1.5 to -3	-1.5 to -3	-1.5 to -3
Pixel size (Å)	0.42	0.42	0.42	0.42	0.42	0.42	0.90
Symmetry imposed	I1	I1	I1	I1	I1	I1	I1
Initial particle images (no.)	386,730	354,088	835,394	449,282	734,883	433,853	112,168
Final particle images (no.)	104,127	56,972	11,999	112,046	81,365	238,315	26,411
Map resolution (Å) FSC threshold: 0.143	2.22	2.20	3.09	2.51	2.18	2.05	3.05
**Refinement:**							
Initial model used	7UD4	7WQP	7UD4	7UD4	7WQP	7UD4	7UD4
Correlation coefficient (CC_{mask})	0.95	0.91	0.92	0.93	0.93	0.91	0.92
**R.M.S devations**							
Bond lengths (Å)	0.003(0)	0.003(0)	0.007(13)	0.002 (0)	0.003 (0)	0.002 (0)	0.005 (0)
Bond angles (˚)	0.501(0)	0.532(0)	1.114(34)	0.459 (0)	0.515(1)	0.398 (0)	0.545 (0)
**Validation**							
MolProbity score	1.03	1.45	1.02	0.73	1.54	1.06	1.21
Clashscore	1.24	2.94	1.11	0.12	2.41	2.48	2.24
Poor rotamers (%)	0.00	0.74	0.22	0	2.4	1.10	0.00
**Ramachandran plot**							
Favored (%)	96.91	94.62	96.75	96.94	96.56	98.26	96.71
Allowed (%)	3.09	5.38	3.25	3.06	3.44	1.74	3.29
Disallowed (%)	0.00	0	0	0.00	0	0	0

Table S2. Average binding kinetic constants of AAVs and AAVR-PKD2

	PKD2 binding affinity rank	Rate of association k_a (1/Ms)	Rate of dissociation k_d (1/s)
X1	1	$1.27 \pm 0.56 \times 10^4$	$0.73 \pm 0.12 \times 10^{-1}$
AAV9	2	$1.17 \pm 0.16 \times 10^4$	$1.32 \pm 0.16 \times 10^{-1}$
PHP.eB	3	$0.86 \pm 0.19 \times 10^4$	$1.19 \pm 0.24 \times 10^{-1}$
X1.1	4	$0.86 \pm 0.2 \times 10^4$	$1.57 \pm 0.29 \times 10^{-1}$
eB.24	5	$0.7 \pm 0.12 \times 10^4$	$1.69 \pm 0.36 \times 10^{-1}$
AAV9-B10	6	$0.52 \pm 0.04 \times 10^4$	$2.44 \pm 0.08 \times 10^{-1}$
CAP-B10	7	$0.55 \pm 0.03 \times 10^4$	$3.35 \pm 0.2 \times 10^{-1}$

Table S3. Sequences of digital droplet PCR (ddPCR) primer and probe sets used for Figure S8

	**Primer & probe sequences**
	**eGFP ddPCR sets**
eGFP forward	CAACAGCCACAACGTCTATATC
eGFP reverse	TGTTCTGCTGGTAGTGGTC
eGFP probe site	AGCAGAAGAACGGCATCAAGGTGA
	**WPRE ddPCR sets**
WPRE forward	TACGCTGCTTTAATGCCTTTG
WPRE reverse	TCCTCATAAAGAGACAGCAACC
WPRE probe site	TCATGCTATTGCTTCCCGTATGGCT
